# Entropic Dynamics Approach to Quantum Electrodynamics

**DOI:** 10.3390/e27121247

**Published:** 2025-12-11

**Authors:** Ariel Caticha

**Affiliations:** Physics Department, University at Albany-SUNY, Albany, NY 12222, USA; acaticha@albany.edu

**Keywords:** entropic dynamics, foundations of quantum mechanics, gauge theory, quantum electrodynamics

## Abstract

Entropic Dynamics (ED) is a framework that allows one to derive quantum theory as a Hamilton–Killing flow on the cotangent bundle of a statistical manifold. These flows are such that they preserve the symplectic and the metric geometries; they explain the linearity of quantum mechanics and the appearance of complex numbers. In this paper the ED framework is extended to deal with local gauge symmetries. More specifically, on the basis of maximum entropy methods and information geometry, for an appropriate choice of ontic variables and constraints, we derive the quantum dynamics of radiation fields interacting with charged particles.

## 1. Introduction

With Einstein’s successful introduction of the concept of photons in 1905, the old enigma of the nature of radiation—is it waves or is it particles?—was brought back into physics [1]. It provided the motivation and the context for de Broglie’s matter waves, for Schrödinger’s wave mechanics, and for Bohr’s complementarity [2]. It also marks the beginning of the long history of controversy over conceptual issues concerning the interpretation of quantum mechanics that endure to this day.

Entropic Dynamics (ED) is a framework that was designed to address these conceptual issues. It provides a *realist* ψ-*epistemic* interpretation that allows the derivation or reconstruction of the mathematical formalism of quantum theory on the basis of established principles of inference, which include probability theory, entropic methods, and information geometry [3] (for a pedagogical review see [4]). Its signal characteristic is a clear distinction of variables that represent the ontology from variables that represent the epistemology. The ontic variables, those which represent what is real, have definite values at all times; however, they are generally unknown and are, therefore, uncertain. Managing these uncertainties requires the introduction of epistemic variables—the probabilities—and it is the evolution of these probabilities that constitutes the main subject of the theory. Thus, ED is quite conservative in that it achieves an ontic/epistemic clarity that is characteristic of classical theories, but, on the other hand, ED is radically non-classical in that the dynamics is totally relegated to the epistemic sector. ED is a dynamics of probabilities; there is no ontic dynamics.

An important consequence of Entropic Dynamics is that if the dynamical variables—the probabilities—are epistemic, then so must be their canonically conjugate momenta. It may be somewhat surprising, but it is nevertheless true that there are no ontic momenta directly associated with either the particles or the potentials. These quantities are absent from the quantum ontology; they emerge only in the classical limit. Thus, ED paints an ontic picture that is radically different from that proposed by classical physics. Furthermore, being a special instance of the updating of probabilities, the dynamics of probabilities is severely constrained by the requirement that it must be consistent with the rules of entropic and Bayesian inference. It is this restriction that motivates the name ‘entropic’ dynamics. Incidentally, it is the interpretation of probabilities as epistemic variables—that probabilities represent degrees of rational belief—that demands a Bayesian approach.

In this paper the framework of Entropic Dynamics is extended to tackle a local gauge theory. We shall apply it to derive a paradigmatic example of quantum electrodynamics (QED). An obvious question immediately arises: Since QED is one of the most successful and most studied theories in physics, why bother? There are two reasons. The first is that questions of interpretation still plague QED. Here, by reconstructing the formalism of QED, we shall clarify issues such as ‘what is ontic?’ and ‘what is epistemic?’. The second reason for tackling QED is to understand how to handle gauge invariance within the context of Entropic Dynamics. The standard approaches to the quantum theory of gauge fields (see, e.g., [5,6,7]) rely heavily on first formulating a classical gauge theory [8], which is subsequently modified by the imposition of quantization rules. ED follows an altogether different approach. It skips the prior formulation of a classical theory and the application of quantization rules and proceeds directly to formulating the quantum theory. The ED approach to quantum gauge fields is conceptually very different from those advocated in the past.

We shall confine our attention to the QED of non-relativistic point charges. This is clearly an idealization, but it will allow us to focus our attention on a number of foundational issues relevant to local gauge theories while avoiding distracting issues (e.g., particle creation, vacuum polarization, etc.) of relativistic origin. However, not all relativistic considerations are irrelevant distractions; some previous work related to the interplay between gauge and relativity effects can be found in [9,10], and other important applications will be left for future work.

This paper is structured as follows: In Section 2 we introduce the microstates that represent the real things, the particles and the gauge fields, recognizing that the representation of the latter is redundant. The symplectic and information geometry of the epistemic phase space of probabilities and their conjugate momenta are established in Section 3. In Section 4 we study kinematics; namely, we characterize those special congruences of curves—the Hamilton–Killing flows—that are adapted to the geometric structures of the epistemic phase space.

Entropic Dynamics is the subject of Section 5. There, we use the method of maximum entropy to calculate the transition probability for an infinitesimally short step and introduce the notion of entropic time as a device to keep track of the accumulation of many such short steps. Then we derive the Hamiltonian that generates evolution in entropic time. As a first, albeit standard, application, we derive the conservation of charge.

In Section 6 we complete the derivation of QED. We discuss the additional requirements that implement gauge invariance and derive the Gauss constraint. Then, we derive the Maxwell equations and verify the emergence of the Coulomb potential. These latter developments yield the already well-known results but with a different interpretation; they serve to reassure us that despite its unorthodox foundations, the ED approach is empirically successful. Section 7 provides a brief summary of the main conclusions, including the fact that in *ED, there are no ontic matter waves and there are no ontic photon particles*.

## 2. The Ontic Microstates

In the ED of nonrelativistic matter, the ontic microstates are assigned properties that are clearly recognized as those of particles: they have definite positions and follow continuous trajectories. On the other hand, the ontic microstates of radiation are assigned properties that are clearly recognized as those of fields: to every point *x* in space one assigns a vector potential degree of freedom $A_{a}\left(x\right)$.

Our system consists of *N* particles and a radiation field living in a flat 3-dimensional Euclidean space X with metric $\delta_{ab}$. The ontic microstates of the particles are represented by the coordinates of their positions $z_{n}^{a}$, collectively denoted by $z=\{z_{n}^{a}\}$. (The index *n*=1…N labels the particles and a=1,2,3 the three spatial coordinates.) The 3N-dimensional ontic configuration space for *N* particles is denoted by $X_{N}$, so that $z\in X_{N}$. The ontic microstates of the radiation field are represented by vector potential distributions $A_{a}\left(x\right)$ with x∈X. To simplify the notation we shall write $A_{a}\left(x\right)=A_{ax}$. The set of field microstates form the ontic configuration space A; each field configuration is a point A∈A. The full ontic configuration space is the Cartesian product $X_{N}A$.

We have already made two important assumptions about the ontology: the particles’ positions and the radiation field have definite values at all times and they follow continuous trajectories. These two assumptions are in contradiction with the standard Copenhagen interpretation of quantum mechanics.

A third and central assumption is close in spirit to the classical theory of gauge fields: the representation of the radiation field by the field $A_{ax}$ is redundant in that a gauge shift to the new field

(1) $$A_{ax}^{\xi }=A_{ax}+\partial_{a}\xi_{x}\;,\;$$

where $\xi_{x}$ is a scalar function, represents the same ontic microstate. (Notation: $\partial_{a}=\partial /\partial x^{a}$. We adopt the usual boundary condition that the fields $A_{ax}$, including the gauge function $\xi_{x}$, vanish at infinity.) Thus, a given ontic microstate of the radiation field can be represented by any member of a whole family of fields $A^{\xi }$ generated by different functions $\xi_{x}$.

The continuity of the paths leads to an important simplification: the dynamics can be studied as a sequence of infinitesimally short steps as the system makes a transition from an initial state (z,A) to a neighboring state $(z^{\prime },A^{\prime })=$(z+Δz,A+ΔA). We seek to develop an Entropic Dynamics that takes into account that the variables $\left(z^{\prime },A^{\prime }\right)$ and $\left(z^{\prime },A^{\prime \xi }\right)$ represent the same ontic microstate.

## 3. The Epistemic Phase Space

The goal of ED is to predict the positions of particles and the values of fields on the basis of information that turns out to be incomplete which forces us to deal with the probability distribution $\rho_{t}\left[z,A\right]$. The notation $\rho_{t}\left[z,A\right]$ is meant to show that ρ is a function of *t* and *z* and is a functional of *A*. The epistemic configuration space, or “e-configuration” space, is the statistical manifold S of normalized gauge-invariant distributions, $\rho \left[z,A^{\xi }\right]=\rho \left[z,A\right]$.

The restrictions to normalized probabilities and to gauge-invariant distributions are serious technical inconveniences that will be provisionally handled by embedding the space S in the larger space

(2) $$S^{+}=\left\{\rho |\;\rho [z,A]\geq 0\right\}$$

of un-normalized probabilities with no gauge symmetry. The appropriate restrictions will be later reintroduced as normalization and gauge constraints on the “physical” states.

The dynamics of probabilities on $S^{+}$ is, however, best defined over the corresponding cotangent bundle $T^{*}S^{+}$, which constitutes the epistemic phase space (or “e-phase” space). We are interested in trajectories in e-phase space, and this leads us to consider those special curves that are naturally adapted to the geometry of $T^{*}S^{+}$. In this section we describe the geometry of $T^{*}S^{+}$ in terms of its symplectic, metric, and complex structures. This is carried out as a straightforward extension to the infinite dimensional case of quantum fields of the corresponding structures that apply to discrete systems [11] and to finite dimensional systems [4]. For a pedagogical background on symplectic and metric geometry, see [12].

First we shall establish some notation. For simplicity the coordinates of a point on $T^{*}S^{+}$ shall be written as $\left(\rho_{zA},\phi_{zA}\right)$, where $\rho_{zA}=\rho \left[z,A\right]$ are coordinates on the base manifold $S^{+}$ and $\phi_{zA}=\phi \left[z,A\right]$ are coordinates on the cotangent space $T_{zA}^{*}S^{+}$. The vector $\bar{V}$ tangent to a generic curve parametrized by λ is written as

(3) $$\bar{V}=\frac{d}{d\lambda }=\int dzDA\left(\frac{d\rho_{zA}}{d\lambda }\frac{\delta }{\delta \rho_{zA}}+\frac{d\phi_{zA}}{d\lambda }\frac{\delta }{\delta \phi_{zA}}\right)=V^{\alpha zA}\frac{\delta }{\delta X^{\alpha zA}}$$

where dz stands for $d^{3N}z$, DA is an appropriate functional measure of integration, and where we introduced the discrete index α=1,2 to stand for ρ and ϕ, respectively.

(4) $$X^{1zA}=\rho_{zA}\;\mathrm{and}\;X^{2zA}=\phi_{zA}\;.\;$$

It is understood that sums and integrations over repeated indices are carried out. The derivative of a functional F[ρ,ϕ] along $\bar{V}$ is

(5) $$\frac{dF}{d\lambda }=\int dzDA\left(\frac{d\rho_{zA}}{d\lambda }\frac{\delta F}{\delta \rho_{zA}}+\frac{d\phi_{zA}}{d\lambda }\frac{\delta F}{\delta \phi_{zA}}\right)=\frac{\delta F}{\delta X^{\alpha zA}}\frac{dX^{\alpha zA}}{d\lambda }=\tilde{\nabla }F\left[\bar{V}\right]\;,$$

where $\tilde{\nabla }$ is the gradient in $T^{*}S^{+}$; that is,

(6) $$\tilde{\nabla }F\left[\cdot \right]=\int dzDA\left(\frac{\delta F}{\delta \rho_{zA}}\tilde{\nabla }\rho_{zA}\left[\cdot \right]+\frac{\delta F}{\delta \phi_{zA}}\tilde{\nabla }\phi_{zA}\left[\cdot \right]\right)=\frac{\delta F}{\delta X^{\alpha zA}}\tilde{\nabla }X^{\alpha zA}\left[\cdot \right]\;,$$

and

(7) $$\tilde{\nabla }X^{1zA}=\tilde{\nabla }\rho_{zA}\;\mathrm{and}\;\tilde{\nabla }X^{2zA}=\tilde{\nabla }\phi_{zA}$$

are the basis covectors. The tilde ‘˜’ is meant to distinguish the gradient $\tilde{\nabla }$ on $T^{*}S^{+}$ from the gradient ∇ on $S^{+}$.

### 3.1. Symplectic Structure

The coordinates $\rho_{zA}$ have a clear meaning that is already explicit for ρ; they represent the *probabilities* of the ontic variables. The interpretation of $\phi_{zA}$ will become clear later, in Section 5.3. These privileged coordinates suggest a natural choice for a symplectic 2-form, namely,

(8) $$\Omega \left[\cdot ,\cdot \right]=\int dzDA\;\left(\tilde{\nabla }\rho_{zA}\left[\cdot \right]⊗\tilde{\nabla }\phi_{zA}\left[\cdot \right]-\tilde{\nabla }\phi_{zA}\left[\cdot \right]⊗\tilde{\nabla }\rho_{zA}\left[\cdot \right]\right)\;,$$

where ⊗ is the tensor product. To find the tensor components of Ω we look at the action of Ω[·,·] on two generic vector fields, $\bar{V}=d/d\lambda$ and $\bar{U}=d/d\mu$. We find

(9) $$\Omega \left[\bar{V},\bar{U}\right]=\int dzDA\;\left[V^{1zA}U^{2zA}-V^{2zA}U^{1zA}\right]=\Omega_{\alpha zA,\beta z^{\prime }A^{\prime }}V^{\alpha zA}U^{\beta z^{\prime }A^{\prime }}\;,\;$$

and the components of Ω, displayed as a matrix, take the familiar form

(10) $$\left[\Omega_{\alpha zA,\beta z^{\prime }A^{\prime }}\right]=\left[\begin{array}{cc} 0 & 1\\ -1 & 0 \end{array}\right]\delta_{\alpha zA,\beta z^{\prime }A^{\prime }}\;,$$

where

(11) $$\delta_{\alpha zA,\beta z^{\prime }A^{\prime }}=\delta_{\alpha \beta }\delta_{zz^{\prime }}\delta_{AA^{\prime }}\;,$$

where $\delta_{\alpha \beta }$ is a Kronecker δ symbol, $\delta_{zz^{\prime }}=\delta \left(z,z^{\prime }\right)$ is the Dirac δ function, and $\delta_{AA^{\prime }}=\delta \left[A,A^{\prime }\right]$ is a Dirac δ functional.

### 3.2. Information Geometry

The simplex of normalized probability distributions is a statistical manifold. Its metric geometry, which turns out to be spherically symmetric, is uniquely determined by information geometry up to a multiplicative overall constant that fixes the units of length (see [4]). The information geometry of the embedding e-configuration space $S^{+}$ of un-normalized probabilities, Equation (2), is also spherically symmetric but it is not uniquely determined. It turns out that without loss of generality (see [4]) the geometries of $S^{+}$ and of the e-phase space $T^{*}S^{+}$ can be assigned the simplest of the spherically symmetric geometries consistent with information geometry, namely, we can choose them to be flat. With this choice the length element on $T^{*}S^{+}$ is

(12) $$\delta {\tilde{\ell }}^{2}=\int dzDA\left[\frac{\hbar }{2\rho_{zA}}\delta \rho_{zA}^{2}+\frac{2\rho_{zA}}{\hbar }\delta \phi_{zA}^{2}\right]=G_{\alpha zA,\beta z^{\prime }A^{\prime }}\delta X^{\alpha zA}\delta X^{\beta z^{\prime }A^{\prime }}\;.$$

Displaying the components of the metric tensor *G* in matrix form, we find

(13) $$\left[G_{zA,z^{\prime }A^{\prime }}\right]=\left[\begin{array}{cc} \frac{\hbar }{2\rho_{zA}} & 0\\ 0 & \frac{2\rho_{zA}}{\hbar } \end{array}\right]\delta_{zA,z^{\prime }A^{\prime }}\;,$$

where *ℏ* is a constant (eventually identified with Planck’s constant) that can be given a geometric interpretation: it controls the relative contributions of $\delta \rho_{zA}$ and $\delta \phi_{zA}$ to the length of the infinitesimal vector (δρ,δϕ).

### 3.3. Complex Structure

The inverse of the metric tensor $G^{-1}$ allows us to raise an index of the symplectic Ω form and define a new tensor *J* with components

(14) $${J^{\alpha zA}}_{\beta z^{\prime }A^{\prime }}=-G^{\alpha zA,\gamma z^{\prime \prime }A^{\prime \prime }}\Omega_{\gamma z^{\prime \prime }A^{\prime \prime },\beta z^{\prime }A^{\prime }}\;.$$

The negative sign is purely conventional. Written as a matrix *J* takes the form

(15) $$\left[{J^{zA}}_{z^{\prime }A^{\prime }}\right]=\left[\begin{array}{cc} 0 & -\frac{2\rho_{zA}}{\hbar }\\ \frac{\hbar }{2\rho_{zA}} & 0 \end{array}\right]\delta_{zA,z^{\prime }A^{\prime }}\;.$$

It is easy to check that

(16) $${J^{\alpha zA}}_{\gamma z^{\prime \prime }A^{\prime \prime }}{J^{\gamma z^{\prime \prime }A^{\prime \prime }}}_{\beta z^{\prime }A^{\prime }}=-\delta_{\alpha zA,\beta z^{\prime }A^{\prime }}\;\mathrm{or}\;J^{2}=-1\;,$$

which means that $T^{*}S^{+}$ has a complex structure and suggests that it might be useful to perform a canonical transformation from the (ρ,ϕ) coordinates to complex coordinates $\left(\psi ,i\hbar \psi^{*}\right)$ where

(17) $$\psi_{zA}=\rho_{zA}^{1/2}e^{i\phi_{zA}/\hbar }\;$$

is the wave functional (or, for simplicity, just the wave “function”). This is the geometrical reason for complex numbers in quantum mechanics: the joint presence of a symplectic and a metric structure implies a complex structure. Such manifolds are usually called Kähler manifolds; the e-phase space $T^{*}S^{+}$ is a very special Kähler manifold in that its metric structure is derived from information geometry. For details of the subtleties of the transformation from (ρ,ϕ) to $\left(\psi ,i\hbar \psi^{*}\right)$ see [4,11].

In wave function coordinates, Equations (12) and (13) become

(18) $$\delta {\tilde{\ell }}^{2}=2\hbar \int dzDA\;\delta \psi_{zA}^{*}\delta \psi_{zA}=2\hbar \langle \delta \psi |\delta \psi \rangle \;,\;\left[G_{zA,z^{\prime }A^{\prime }}\right]=-i\left[\begin{array}{cc} 0 & 1\\ 1 & 0 \end{array}\right]\delta_{zA,z^{\prime }A^{\prime }}\;.$$

The transformation (17) is canonical in the sense that the components of the symplectic tensor Ω, Equation (10), remain unchanged. Notice also that, unlike the components of the metric tensor *G* in (ρ,ϕ) coordinates (Equation (13)), in wave function coordinates the components of *G* (Equation (18)) are constants, which makes explicit both the flatness of the e-phase space $T^{*}S^{+}$ and the convenience of introducing wave functions.

## 4. Kinematics

### 4.1. Hamilton Flows

Given the symplectic form Ω, there are families of vector fields $\bar{H}$ that are special in the sense that the Lie derivative of Ω along $\bar{H}$ vanishes.

(19) $${£}_{\bar{H}}\Omega =0\;,$$

that is, the symplectic form Ω is preserved along the integral curves generated by $\bar{H}$. These curves, which are naturally adapted to the symplectic structure of $T^{*}S^{+}$, are such that associated with the vector field $\bar{H}$ there exists a scalar function $\tilde{H}$ such that the components of $\bar{H}$ are given by Hamilton’s equations [12]. In wave function coordinates we have

(20) $$H^{1zA}=\frac{d\psi_{zA}}{d\tau }=\frac{1}{i\hbar }\frac{\delta \tilde{H}}{\delta \psi_{zA}^{*}}\;\mathrm{and}\;H^{2zA}=i\hbar \frac{d\psi_{zA}^{*}}{d\tau }=-\frac{\delta \tilde{H}}{\partial \psi_{zA}}\;,$$

where τ is the parameter along the curves. This justifies calling $\bar{H}$ the *Hamiltonian vector field* associated with the *Hamiltonian function* $\tilde{H}$, and the congruence of these curves is called a Hamilton flow. Eventually we shall choose $\tilde{H}$ to be the actual Hamiltonian that generates a flow in time. At this point, however, $\tilde{H}$ is some generic Hamiltonian function, and there is no implication that the parameter τ represents time; for example, $\tilde{H}$ could be the generator of rotations and τ the corresponding rotation angle.

The action of Ω[·,·] on two Hamiltonian vector fields, $\bar{V}=d/d\lambda$ and $\bar{U}=d/d\mu$, generated, respectively, by $\tilde{V}$ and $\tilde{U}$, is

(21) $$\Omega \left(\bar{V},\bar{U}\right)=\int dzDA\;i\hbar \left(\frac{d\psi_{zA}}{d\lambda }\frac{d\psi_{zA}^{*}}{d\mu }-\frac{d\psi_{zA}^{*}}{d\lambda }\frac{d\psi_{zA}}{d\mu }\right)\;,$$

which, using (20), gives

(22) $$\Omega \left(\bar{V},\bar{U}\right)=\int dzDA\frac{1}{i\hbar }\left(\frac{\delta \tilde{V}}{\delta \psi_{zA}}\frac{\delta \tilde{U}}{\delta \psi_{zA}^{*}}-\frac{\delta \tilde{V}}{\delta \psi_{zA}^{*}}\frac{\delta \tilde{U}}{\delta \psi_{zA}}\right)\overset{\mathrm{def}}{=}\left\{\tilde{V},\tilde{U}\right\}\;,$$

which is recognized as the Poisson bracket.

### 4.2. Normalization

We shall eventually require that probabilities be normalized. This is expressed as a constraint,

(23) $$\tilde{N}=0\;\mathrm{where}\;\tilde{N}=1-\int dzDA\;\psi_{zA}^{*}\psi_{zA}\;,$$

on the physically relevant states. Since the constraint $\tilde{N}=\mathrm{const}$ must be preserved by time evolution we shall require Hamiltonian functions such that

(24) $$\frac{d\tilde{N}}{d\tau }=\left\{\tilde{N},\tilde{H}\right\}=0\;.$$

Conversely, if $\tilde{N}$ is conserved along the flow generated by $\tilde{H}$, then $\tilde{H}$ is conserved along the Hamilton flow generated by $\tilde{N}$ and parametrized by ν,

(25) $$\frac{d\tilde{H}}{d\nu }=\left\{\tilde{H},\tilde{N}\right\}=0\;,$$

which means that $\tilde{N}$ generates symmetry. The flow generated by $\tilde{N}$ is given by Hamilton’s equation,

(26) $$\frac{d\psi_{zA}}{d\nu }=\frac{1}{i\hbar }\frac{\delta \tilde{N}}{\delta \psi_{zA}^{*}}=\frac{i}{\hbar }\psi_{zA}\;,$$

which is easily integrated to give

(27) $$\psi_{zA}\left(\nu \right)=\psi_{zA}\left(0\right)e^{i\nu /\hbar }\;.$$

The interpretation is as follows. The symmetry generated by $\tilde{N}$ is the consequence of embedding the e-phase space $T^{*}S$ of normalized probabilities into the larger embedding space $T^{*}S^{+}$ of un-normalized probabilities. One consequence of introducing one superfluous coordinate ρ is also introducing a superfluous momentum ϕ. The extra coordinate ρ is eliminated by imposing the constraint $\tilde{N}=0$. The extra momentum ϕ is eliminated by declaring it unphysical. This is achieved by declaring that states along the flow are equivalent, that is, states $\psi_{zA}\left(\nu \right)$ with different ν lie on the same “ray”.

### 4.3. Hamilton–Killing Flows

We can further restrict the choice of the Hamiltonian function $\tilde{H}$ by requiring that, in addition to preserving the symplectic structure Ω, it must also preserve the metric structure *G*. Thus we seek a Hamiltonian $\tilde{H}$ that satisfies

(28) $${£}_{\bar{H}}\Omega =0\;\mathrm{and}\;{£}_{\bar{H}}G=0\;,$$

and generates flows that are simultaneously Hamilton flows and Killing flows. As shown in [11] (see also [4]), the Hamilton–Killing flows (or HK flows) are generated by Hamiltonians of the form

(29) $$\tilde{H}\left[\psi ,\psi^{*}\right]=\int dzDAdz^{\prime }DA^{\prime }\;\psi_{zA}^{*}{\hat{H}}_{zA,z^{\prime }A^{\prime }}\psi_{z^{\prime }A^{\prime }}+\int dzDA\left(\;\psi_{zA}^{*}{\hat{L}}_{zA}+{\hat{M}}_{zA}\psi_{zA}\right)\;,$$

where the kernels $\hat{H}$, $\hat{L}$, and $\hat{M}$ are independent of $\psi^{*}$ and ψ. Requiring that the flow also preserve normalization implies that $\tilde{H}$ must be invariant under the global phase shift (27). Therefore, ${\hat{L}}_{zA}=0$ and ${\hat{M}}_{zA}=0$, and $\tilde{H}$ must be bilinear in $\psi_{zA}$ and $\psi_{zA}^{*}$; that is,

(30) $$\tilde{H}\left[\psi ,\psi^{*}\right]=\int dzDAdz^{\prime }DA^{\prime }\;\psi_{zA}^{*}{\hat{H}}_{zA,z^{\prime }A^{\prime }}\psi_{z^{\prime }A^{\prime }}\;.$$

The kernel ${\hat{H}}_{zA,z^{\prime }A^{\prime }}$ can be conveniently interpreted as the matrix element,

(31) $${\hat{H}}_{zA,z^{\prime }A^{\prime }}=\langle zA|\hat{H}|z^{\prime }A^{\prime }\rangle \;,$$

of a self-adjoint operator $\hat{H}$. (For a pedagogical presentation of the Schrödinger representation in quantum field theory, see [13].) Such bilinear Hamiltonians lead to equations of motion,

(32) $$i\hbar \frac{d\psi_{zA}}{d\tau }=\frac{\delta \tilde{H}}{\delta \psi_{zA}^{*}}=\int dz^{\prime }DA^{\prime }{\hat{H}}_{zA,z^{\prime }A^{\prime }}\psi_{z^{\prime }A^{\prime }}\;,$$

that are recognized as Schrödinger equations. Thus, the requirement of HK flows that also preserve normalization provides an explanation for the linearity of quantum mechanics and its attendant superposition principle.

The main result of this section is that the special curves that are adapted to the symplectic and the information metric structures of the e-phase $T^{*}S^{+}$ are HK flows generated by bilinear Hamiltonians. This concludes our discussion of kinematics.

## 5. Entropic Dynamics

Having figured that HK flows yield specially privileged families of curves, our next goal is to figure out which of these curves are good candidates to be actual trajectories. We want to derive the dynamics of probabilities. First, we use the method of maximum entropy to calculate the transition probability of a short step; this is the central idea behind any *entropic* dynamics. Then, since the rules of inference are atemporal, the assumptions behind the construction of the concept of time must be explicitly stated. An *entropic* notion of time is introduced as a book-keeping device to keep track of the accumulation of a succession of short steps. The final step yields a Hamiltonian that generates an HK flow in entropic time and allows us to write the Schrödinger functional equation. Similar ideas have been previously developed in [4,11]. Here the ED framework will be further extended to the case of a field with local gauge symmetry.

### 5.1. The Transition Probability for a Short Step

The continuity of trajectories in the ontic configuration space allows us to analyze the evolution as a sequence of short steps, which leads us to calculate the transition probability $P\left[z^{\prime }A^{\prime }|zA\right]=P_{z^{\prime }A^{\prime }|zA}$ from *z* to $z^{\prime }=z+\Delta z$ and from *A* to $A^{\prime }=A+\Delta A$. To guarantee that the dynamics is consistent with the rules for updating probabilities the transition probability $P_{z^{\prime }{\hat{A}}^{\prime }|zA}$ is found by maximizing the entropy

(33) $$S\left[z^{\prime }A^{\prime }|zA\right]=-\int dz^{\prime }DA^{\prime }\;P_{z^{\prime }A^{\prime }|zA}\log\frac{P_{z^{\prime }A^{\prime }|zA}}{Q_{z^{\prime }A^{\prime }|zA}}\;$$

relative to the prior *Q* and subject to the appropriate constraints. The relevant physical information is introduced in two different ways. The information that is common to all short steps is codified into the prior $Q_{z^{\prime }A^{\prime }|zA}$ while the information that is specific to each individual short step is introduced as a constraint on the transition probability $P_{z^{\prime }A^{\prime }|zA}$.

The prior *Q* codifies the information that the particles and the field evolve by taking infinitesimally short steps, but *Q* is otherwise maximally uninformative in the sense that it must reflect the translational and rotational invariance of the space X and it must express total ignorance about any correlations. The desired prior is Gaussian,

(34) $$Q_{z^{\prime }A^{\prime }|zA}\propto \exp-\frac{1}{2}\left[\sum_{n}\alpha_{n}\delta_{ab}\Delta z_{n}^{a}\Delta z_{n}^{b}+\alpha \int d^{3}x\;\delta^{ab}\Delta A_{ax}\Delta A_{bx}\right]\;.$$

where the constants $\alpha_{n}$ and α are Lagrange multipliers to be specified later. (The Gaussian prior *Q* can itself be derived using the method of maximum entropy.) The multipliers $\alpha_{n}$ and α will eventually be made arbitrarily large to ensure that *Q* leads to intrinsic changes that are infinitesimally small. The index *n* in $\alpha_{n}$ indicates that the particles need not be identical.

The physical information about directionality and correlations is specific to each short step. It is introduced via a constraint that involves a functional $\varphi \left[z,A\right]=\varphi_{zA}$ called the “drift potential.” The constraint consists of imposing that the expected change of $\varphi_{zA}$ over a short step is some small amount $\kappa^{\prime }$,

(35) $$\langle \Delta \varphi_{zA}\rangle =\int dz^{\prime }DA^{\prime }\;P_{z^{\prime }A^{\prime }|zA}\;\Delta \varphi_{zA}=\kappa^{\prime }\;.$$

More explicitly, we can Taylor-expand $\Delta \varphi_{zA}$ and write

(36) $$\int dz^{\prime }DA^{\prime }\;P_{z^{\prime }A^{\prime }|zA}\left[\sum_{n}\frac{\partial \varphi_{zA}}{\partial z_{n}^{a}}\Delta z_{n}^{a}+\int d^{3}x\;\frac{\delta \varphi_{zA}}{\delta A_{ax}}\Delta A_{ax}\right]=\kappa^{\prime }\;.$$

As in other forms of ED, the drift potential $\varphi_{zA}$ is of central importance. It plays three separate roles: (1) it serves to express a constraint that codifies physical information about short steps; (2) it turns out to be a momentum that is canonically conjugate to the probability $\rho_{zA}$; and (3) it will be closely related to the phase $\phi_{zA}$ of the wave functional $\psi_{zA}$ in Equation (17).

Finally, we require one last set of constraints that describes how each particle interacts with the gauge field *A*. We impose that the expected displacement $\langle \Delta z_{n}^{a}\rangle$ for each particle satisfies

(37) $$\langle \Delta z_{n}^{a}\rangle A_{az_{n}}=\kappa_{n}^{\prime \prime }\;\mathrm{for}\;n=1\ldots N\;.$$

The coupling is local: the particle at *z* couples to the field at *z*. The numerical values of the quantities $\kappa^{\prime }$ and $\kappa_{n}^{\prime \prime }$ could be specified directly, but it is more convenient to specify them indirectly in terms of the corresponding Lagrange multipliers.

We are now ready to assign the transition probability $P_{z^{\prime }A^{\prime }|zA}$. Maximize the entropy (33) relative to the prior (34) and subject to the constraints (36) and (37), and normalization. The result is a Gaussian distribution,

(38) $$\begin{array}{cc} P_{z^{\prime }A^{\prime }|zA} & \propto \exp\sum_{n}\left(-\frac{\alpha_{n}}{2}\delta_{ab}\Delta z_{n}^{a}\Delta z_{n}^{b}+\alpha^{\prime }\left(\partial_{na}\varphi_{zA}-\beta_{n}A_{az_{n}}\right)\Delta z_{n}^{a}\right)\\ \; & \times \exp\int d^{3}x\;\left(-\frac{\alpha }{2}\delta^{ab}\Delta A_{ax}\Delta A_{bx}+\alpha^{\prime }\frac{\delta \varphi_{zA}}{\delta A_{ax}}\Delta A_{ax}\right)\;, \end{array}$$

where $\alpha_{n}$, α, $\alpha^{\prime }$, and $\alpha^{\prime }\beta_{n}$ are Lagrange multipliers, and $\partial_{na}=\partial /\partial z_{n}^{a}$. Later we will write the $\beta_{n}$ parameters as $\beta_{n}=q_{n}/c$, where $q_{n}$ will be identified with the electric charges in Heaviside–Lorentz units (i.e., rationalized Gaussian units), and *c* is the speed of light. Completing the square in the exponent, *P* can be rewritten as

(39) $$\begin{array}{cc} P_{z^{\prime }A^{\prime }|zA} & \propto \exp-\frac{1}{2}\sum_{n}\alpha_{n}\delta_{ab}\left(\Delta z_{n}^{a}-{\bar{\Delta z}}_{n}^{a}\right)\left(\Delta z_{n}^{b}-{\bar{\Delta z}}_{n}^{b}\right)\\ \; & \left.+\int d^{3}x\;\alpha \delta^{ab}\left(\Delta A_{ax}-{\bar{\Delta A}}_{ax}\right)\left(\Delta A_{bx}-{\bar{\Delta A}}_{bx}\right)\right] \end{array}$$

where ${\bar{\Delta z}}_{n}^{a}$ and ${\bar{\Delta A}}_{ax}$ are the expected steps,

(40) $$\langle \Delta z_{n}^{a}\rangle ={\bar{\Delta z}}_{n}^{a}=\frac{\alpha^{\prime }}{\alpha_{n}}\delta^{ab}\left(\partial_{nb}\varphi_{zA}-\beta_{n}A_{bz_{n}}\right)\;,$$

(41) $$\langle \Delta A_{ax}\rangle ={\bar{\Delta A}}_{ax}=\frac{\alpha^{\prime }}{\alpha }\delta_{ab}\frac{\delta \varphi_{zA}}{\delta A_{bx}}\;.$$

Let a generic step be described as

(42) $$\Delta z_{n}^{a}={\bar{\Delta z}}_{n}^{a}+\Delta w_{n}^{a}\;\mathrm{and}\;\Delta A_{ax}={\bar{\Delta A}}_{ax}+\Delta W_{ax}\;.$$

Then the fluctuations $\Delta w_{n}^{a}$ and $\Delta W_{ax}$ satisfy

(43) $$\begin{array}{cc} \langle \Delta w_{n}^{a}\rangle & =0\;\;\mathrm{and}\;\langle \Delta w_{n}^{a}\Delta w_{n^{\prime }}^{b}\rangle =\frac{1}{\alpha_{n}}\delta_{ab}\delta_{nn^{\prime }}\;, \end{array}$$

(44) $$\begin{array}{cc} \langle \Delta W_{ax}\rangle & =0\;\mathrm{and}\;\langle \Delta W_{ax}\Delta W_{bx^{\prime }}\rangle =\frac{1}{\alpha }\delta_{ab}\delta_{xx^{\prime }}\;, \end{array}$$

while the correlations vanish:

(45) $$\langle \Delta z_{n}^{a}\Delta A_{bx}\rangle =\langle \Delta z_{n}^{a}\rangle \langle \Delta A_{bx}\rangle \;\mathrm{and}\;\langle \Delta w_{n}^{a}\Delta W_{bx}\rangle =\langle \Delta w_{n}^{a}\rangle \langle \Delta W_{bx}\rangle =0\;.$$

### 5.2. Entropic Time

An Entropic Dynamics of probabilities requires an entropic notion of time. This involves introducing the concept of instants that are suitably ordered and adopting a convenient measure of the interval between instants. In ED an instant is specified by the information—codified into the functions $\rho_{zA}$ and $\varphi_{zA}$—that is sufficient for generating the next instant. Indeed, if the distribution ρ and the drift potential φ refer to the instant *t*, then the distribution

(46) $$\rho_{z^{\prime }A^{\prime }}^{\prime }=\int dzDA\;P_{z^{\prime }A^{\prime }|zA}\rho_{zA}$$

generated from $\rho_{zA}$ by $P_{z^{\prime }{\hat{A}}^{\prime }|zA}$ serves both to define the dynamics of ρ and to construct what we mean by the “next” instant $t^{\prime }$. Later we shall complete the construction by specifying the evolution of $\varphi_{zA}$.

Before proceeding further, a couple of remarks are in order. In Equation (46) we see that given the information codified into a present instant *t*, the future instant $t^{\prime }>t$ turns out to be independent of any past instant $t^{\prime \prime }<t$. Thus, formally, ED is a Markovian process. However, it is important to emphasize that there is an important difference. Markovian processes are assumed to develop in an already existing time determined by external clocks. In ED there is no such pre-existing time; information about the system at one instant is used to generate or construct the next instant. Thus, ED is fully relational with respect to time [14].

The second remark is that the evolution equation involves a transition probability that was derived by maximizing an entropy which implies that there is a clear evolution from a past instant towards a future instant. Therefore, in ED the instants are ordered and there is a natural arrow of time.

We shall now address the unfinished task of specifying the Lagrange multipliers that appear in the transition probability, Equation (38). As discussed in [4] the last step in the construction of time is specifying the measure of duration— the interval Δt between successive instants—in terms of the multipliers $\alpha_{n}$, α, and $\alpha^{\prime }$. Thus, *the transition probability provides us with a clock*. As a matter of convenience we choose multipliers adapted to the translational symmetries of Newtonian space and, accordingly, we shall choose them to be constants independent of *x* and t.

Furthermore, we want the transition probability to lead to infinitesimally short steps and particles with well-defined expected velocities. Equation (40) shows that this is achieved choosing the ratio $\alpha^{\prime }/\alpha_{n}$ proportional to Δt,

(47) $$\frac{\alpha^{\prime }}{\alpha_{n}}=\frac{1}{m_{n}}\Delta t\;,$$

where we introduced proportionality constants $m_{n}$ that will eventually be identified with the particle masses. As in previous work we shall also take $\alpha^{\prime }=$1/η to be constant and set η such that if Δt has units of time, then $m_{n}$ has units of mass. Then,

(48) $$\alpha^{\prime }=\frac{1}{\eta }\;\mathrm{so}\;\mathrm{that}\;\alpha_{n}=\frac{m_{n}}{\eta \Delta t}\;.$$

The expected drift for particles, Equation (40), and their fluctuations, Equation (43), become

(49) $${\bar{\Delta z}}_{n}^{a}=\frac{\Delta t}{m_{n}}\delta^{ab}\left(\partial_{nb}\varphi_{zA}-\beta_{n}A_{bz_{n}}\right)\;\mathrm{and}\;\langle \Delta w_{n}^{a}\Delta w_{n^{\prime }}^{b}\rangle =\Delta t\frac{\eta }{m_{n}}\delta^{ab}\delta_{nn^{\prime }}\;.$$

As far as the radiation goes, its multiplier α is also chosen to lead to a well-defined expected rate of change,

(50) $$\alpha =\frac{1}{c^{2}\eta \Delta t}\;.$$

The proportionality constant α is such that the field *A* is measured in Heaviside–Lorentz units. Then,

(51) $${\bar{\Delta A}}_{ax}=\Delta t\;c^{2}\delta_{ab}\frac{\delta \varphi_{zA}}{\delta A_{bx}}\;\mathrm{and}\;\langle \Delta W_{ax}\Delta W_{bx^{\prime }}\rangle =\Delta t\;c^{2}\eta \delta_{ab}\delta_{xx^{\prime }}\;.$$

### 5.3. The Hamiltonian

Our next goal is to identify a Hamiltonian $\tilde{H}\left[\rho ,\phi \right]$ that generates evolution in entropic time. The traditional approach in mechanics makes use of a Lagrangian $L(q,\dot{q})$ to identify the momentum $p=\partial L/\partial \dot{q}$ that is conjugate to the coordinate *q*. In ED no Lagrangians are available, and an alternative method is required. We propose that a momentum conjugate to $\rho_{zA}$ can be identified from Equation (46), which is the evolution equation for ρ written in integral form. The process takes a few steps. The first step consists of rewriting Equation (46) in differential form:

(52) $$\frac{\partial \rho_{zA}}{\partial t}=-\sum_{n}\frac{\partial }{\partial z_{n}^{a}}\left(v_{n}^{a}\rho_{zA}\right)-\int d^{3}x\;\frac{\delta }{\delta A_{ax}}\left(u_{ax}\rho_{zA}\right)\;.$$

This is a continuity equation where the velocity of the probability flow—the current velocity—has components along $z_{n}^{a}$ and along $A_{ax}$ given by

(53) $$v_{n}^{a}\left[z,A\right]=\frac{\delta^{ab}}{m_{n}}\left(\partial_{nb}\phi_{zA}-\beta_{n}A_{bz_{n}}\right)\;\mathrm{and}\;u_{ax}\left[z,A\right]=c^{2}\delta_{ab}\frac{\delta \phi_{zA}}{\delta A_{bx}}\;,$$

where

(54) $$\phi_{zA}=\varphi_{zA}-\eta \log\rho_{zA}^{1/2}\;.\;$$

The derivation leading from (46) to (52) is given in Appendix A. The second step is to show that (52) can be written in Hamiltonian form,

(55) $$\frac{\partial \rho_{zA}}{\partial t}=\frac{\delta \tilde{H}}{\delta \phi_{zA}}\;,$$

which suggests that $\phi_{zA}$ is a convenient canonical momentum. Equation (54) shows that $\phi_{zA}$ and $\varphi_{zA}$ differ by a function of the generalized coordinate $\rho_{zA}$. This is just a canonical transformation and the choice of either $\varphi_{zA}$ or $\phi_{zA}$ as the conjugate momentum is just a matter of convenience. To show that (52) can be written in the form (55) we must find the Hamiltonian $\tilde{H}$. This is straightforward: equating (52) to (55) yields a linear functional differential equation that can be easily integrated for $\tilde{H}$. The result is

(56) $$\begin{array}{cc} \tilde{H}\left[\rho ,\phi \right] & =\int dzDA\;\rho_{zA}\left[\sum_{n}\frac{\delta^{ab}}{2m_{n}}\left(\partial_{na}\phi_{zA}-\beta_{n}A_{az_{n}}\right)\left(\partial_{nb}\phi_{zA}-\beta_{n}A_{bz_{n}}\right)\right.\\ \; & +\int d^{3}x\;\frac{c^{2}}{2}\delta_{ab}\frac{\delta \phi_{zA}}{\delta A_{ax}}\frac{\delta \phi_{zA}}{\delta A_{bx}}+\tilde{F}\left[\rho \right] \end{array}$$

where the unspecified functional $\tilde{F}\left[\rho \right]$ is an integration constant. We write $\tilde{F}\left[\rho \right]$ to emphasize that $\tilde{F}$ is independent of ϕ, but in principle we could have $\tilde{F}=\tilde{F}\left[\rho ;z,A,t\right]$. It is straightforward to check that (56) is a solution to (55): just take a variation $\delta \phi_{zA}$ and integrate by parts.

Yet another piece of information that guides our choice of Hamiltonian is that in order to generate HK flows $\tilde{H}$ must take the bilinear form, Equation (30). Transforming to wave function coordinates, Equation (56) becomes

(57) $$\tilde{H}=\int dzDA\;\psi_{zA}^{*}\left(\sum_{n}\frac{-\hbar^{2}}{2m_{n}}\delta^{ab}D_{na}D_{nb}-\int d^{3}x\;\frac{{\left(\hbar c\right)}^{2}}{2}\delta_{ab}\frac{\delta^{2}}{\delta A_{ax}\delta A_{bx}}\right)\psi_{zA}+\tilde{V}\left[\rho \right]$$

where, setting $\beta_{n}=q_{n}/c$, $D_{na}$ is the covariant derivative,

(58) $$D_{na}=\frac{\partial }{\partial z_{n}^{a}}-i\frac{q_{n}}{\hbar c}A_{a}\left(z_{n}\right)\;,$$

and the new functional $\tilde{V}\left[\rho \right]$ differs from the old $\tilde{F}\left[\rho \right]$ by a functional of ρ,

(59) $$\tilde{V}\left[\rho \right]=\tilde{F}\left[\rho \right]-\int dzDA\;\left(\sum_{n}\frac{\hbar^{2}}{8m_{n}}\frac{\delta^{ab}}{\rho_{zA}}\frac{\partial \rho_{zA}}{\partial z_{n}^{a}}\frac{\partial \rho_{zA}}{\partial z_{n}^{b}}+\int d^{3}x\;\frac{{\left(\hbar c\right)}^{2}}{8}\frac{\delta_{ab}}{\rho_{zA}}\frac{\delta \rho_{zA}}{\delta A_{ax}}\frac{\delta \rho_{zA}}{\delta A_{bx}}\right)\;.$$

In order to generate an HK flow the functional $\tilde{V}\left[\rho \right]$ must itself be bilinear,

(60) $$\tilde{V}\left[\psi ,\psi^{*}\right]=\int dz^{\prime }DA^{\prime }dz^{\prime \prime }DA^{\prime \prime g}\;\psi_{z^{\prime }A^{\prime }}^{*}{\hat{V}}_{z^{\prime }A^{\prime },z^{\prime \prime }A^{\prime \prime }}\psi_{z^{\prime \prime }A^{\prime \prime }}\;,$$

for some Hermitian kernel $\hat{V}$, while still remaining independent of ϕ,

(61) $$\frac{\delta \tilde{V}\left[\psi ,\psi^{*}\right]}{\delta \phi_{zA}}=\frac{\delta \tilde{V}\left[\rho \right]}{\delta \phi_{zA}}=0\;.$$

Substituting (60) into (61) leads to

(62) $$\int dz^{\prime }DA^{\prime }dz^{\prime \prime }DA^{\prime \prime }\;\left(-\delta_{z^{\prime }A^{\prime },zA}+\delta_{z^{\prime \prime }A^{\prime \prime },zA}\right)\psi_{z^{\prime }A^{\prime }}^{*}{\hat{V}}_{z^{\prime }A^{\prime },z^{\prime \prime }A^{\prime \prime }}\psi_{z^{\prime \prime }A^{\prime \prime }}=0\;,$$

which must be satisfied for all choices of $\psi_{z^{\prime }A^{\prime }}^{*}$ and $\psi_{z^{\prime \prime }A^{\prime \prime }}$. Therefore the kernel ${\hat{V}}_{z^{\prime }A^{\prime },z^{\prime \prime }A^{\prime \prime }}$ must be local in the ontic configuration space $X_{N}A$,

(63) $${\hat{V}}_{z^{\prime }A^{\prime },z^{\prime \prime }A^{\prime \prime }}=\delta_{z^{\prime }A^{\prime },z^{\prime \prime }A^{\prime \prime }}{\hat{V}}_{z^{\prime }A^{\prime }}\;,$$

where ${\hat{V}}_{zA}$ is a real functional that plays the role of a potential. This is how potentials are introduced in the ED framework. Substituting (63) into (57), we obtain the Hamiltonian

(64) $$\tilde{H}=\int dzDA\;\psi_{zA}^{*}\hat{H}\psi_{zA}\;,$$

where the kernel $\hat{H}$ is the operator

(65) $$\hat{H}\overset{\mathrm{def}}{=}\sum_{n}\frac{-\hbar^{2}}{2m_{n}}\delta^{ab}D_{na}D_{nb}+\int d^{3}x\;\frac{{\left(\hbar c\right)}^{2}}{2}\delta_{ab}\frac{\delta^{2}}{\delta A_{ax}\delta A_{bx}}+{\hat{V}}_{zA}\;.$$

For brevity we will refer to both $\tilde{H}$ and $\hat{H}$ as “the Hamiltonian”. The Schrödinger equation is the Hamilton equation of motion, Equation (32):

(66) $$\frac{d\psi_{zA}}{dt}=\frac{1}{i\hbar }\frac{\delta \tilde{H}}{\delta \psi_{zA}^{*}}\;\mathrm{or}\;i\hbar \frac{d\psi_{zA}}{dt}={\hat{H}}_{zA}\psi_{zA}\;.$$

A familiar example is the potential ${\hat{V}}_{zA}$ for the pure electrodynamics of radiation and particles in a vacuum,

(67) $${\hat{V}}_{zA}=\int d^{3}x\;\frac{1}{2}\delta_{ab}{\hat{B}}_{x}^{a}{\hat{B}}_{x}^{b}\;\mathrm{where}\;{\vec{B}}_{x}\overset{\mathrm{def}}{=}{\vec{\partial }}_{x}\times {\vec{A}}_{x}\;,$$

Additional terms may be included to describe direct particle–particle interactions and the index of refraction or other effects of the dielectric polarization of a material medium.

### 5.4. Charge Conservation

At this point in the argument, we do not yet have a locally gauge invariant dynamics; this will depend on appropriate choices of the potential ${\hat{V}}_{zA}$ and of the initial wave function $\psi (t_{0})$. However, irrespective of the choice of ${\hat{V}}_{zA}$ and $\psi (t_{0})$, there is a symmetry under the global phase transformation

(68) $$\psi_{zA}\to \psi_{zA}^{\lambda }=\psi_{zA}e^{i\lambda }\;\mathrm{and}\;A_{ax}\to A_{ax}^{\lambda }=A_{ax}$$

where λ is a constant. One expects that there is a conservation law associated with this symmetry and, indeed, there is. The derivation can be carried out following any of several standard methods. We shall make use Hamilton’s Equation (55), which is the continuity Equation (52).

The operators that represent the densities of electric charge and electric current are

(69) $${\hat{\rho }}_{x}^{e}\left[z,A\right]=\sum_{n}q_{n}\delta_{z_{n}x}\;\mathrm{and}\;{\hat{J}}_{x}^{ea}\left[z,A\right]=\sum_{n}q_{n}\delta_{z_{n}x}v_{n}^{a}\;.$$

The corresponding Hamiltonian functionals are

(70) $${\tilde{\rho }}_{x}^{e}=\int dzDA\;\psi_{zA}^{*}{\hat{\rho }}_{x}^{e}\psi_{zA}=\int dzDA\;\rho_{zA}\sum_{n}q_{n}\delta_{z_{n}x}\;,$$

and

(71) $${\tilde{J}}_{x}^{ea}=\int dzDA\;\psi_{zA}^{*}{\hat{J}}_{x}^{ea}\psi_{zA}=\int dzDA\;\rho_{zA}\sum_{n}q_{n}\delta_{z_{n}x}v_{n}^{a}\;.$$

Using Equation (52) we find

$$\begin{array}{cc} \frac{\partial {\tilde{\rho }}_{x}^{e}}{\partial t} & =\int dzDA\;{\hat{\rho }}_{x}^{e}\frac{\partial \rho_{zA}}{\partial t}=\\ \; & =-\int dzDA\sum_{n^{\prime }}q_{n^{\prime }}\delta_{z_{n^{\prime }}x}\left(\sum_{n}\frac{\partial }{\partial z_{n}^{a}}\left(v_{n}^{a}\rho_{zA}\right)+\int d^{3}x\;\frac{\delta }{\delta A_{ax}}\left(u_{ax}\rho_{zA}\right)\right) \end{array}$$

The integral over DA of the last term is a vanishing surface term,

(72) $$\int DA\;\frac{\delta }{\delta A_{ax}}\left(u_{ax}\rho_{zA}\right)=0\;.$$

Therefore

$$\frac{\partial {\tilde{\rho }}_{x}^{e}}{\partial t}=-\int dzDA\sum_{n^{\prime }n}q_{n^{\prime }}\delta_{z_{n^{\prime }}x}\frac{\partial }{\partial z_{n}^{a}}\left(v_{n}^{a}\rho_{zA}\right)\;.$$

Integrating by parts and using

(73) $$\sum_{n^{\prime }}q_{n^{\prime }}\frac{\partial }{\partial z_{n}^{a}}\delta \left({\vec{z}}_{n^{\prime }}-\vec{x}\right)=q_{n}\frac{\partial }{\partial z_{n}^{a}}\delta \left({\vec{z}}_{n}-\vec{x}\right)=-q_{n}\frac{\partial }{\partial x^{a}}\delta \left({\vec{z}}_{n}-\vec{x}\right)\;,$$

we find

$$\frac{\partial {\tilde{\rho }}_{x}^{e}}{\partial t}=-\frac{\partial }{\partial x^{a}}\int dzDA\;\sum_{n}q_{n}\delta_{z_{n}x}v_{n}^{a}\rho_{zA}\;,$$

which, using (71), yields the desired result,

(74) $$\frac{\partial }{\partial t}{\tilde{\rho }}_{x}^{e}=-\frac{\partial }{\partial x^{a}}{\tilde{J}}_{x}^{ea}\;.$$

We have derived a mathematically legitimate conservation law with a perhaps unfamiliar interpretation and this warrants some comments. First, as is evident from (70) and (71), ${\tilde{\rho }}_{x}^{e}$ and ${\tilde{J}}_{x}^{ea}$ are expected values. We claim that we have a charge conservation law because the identity (74) holds for any arbitrary choice of the probability ρ[z,A], but the electric charges $q_{n}$ are not some ontic substance that is physically attached to point particles. They are parameters that operate at the epistemic level of probabilities; they are Lagrange multipliers that regulate the probabilistic correlations between particles and fields. Indeed, as befits an Entropic Dynamics of probabilities, the velocity $v_{n}^{a}\left[z,A\right]$ that defines the electric current is not the velocity of the particles but the velocity of the probability flow.

The fact that charges (and also masses) are introduced via Lagrange multipliers leads us to a second comment motivated by a potential analogy with the statistical mechanics of thermal equilibrium. The point is that quantities such as temperatures and chemical potentials are also introduced via Lagrange multipliers and they are subject to fluctuations, which raises the question of whether charges and masses might also fluctuate. In principle the answer is yes, but it is not clear how to bring about such an effect, and perhaps the analogy, like all analogies, should not be pushed too far.

In the case of thermal equilibrium, the precise specification of the expected energy implies a precise value of temperature. To induce temperature fluctuations one requires an uncertainty in the energy constraint which is brought about by spontaneous energy exchanges with an environment. Similarly, in ED the precise specification of expected value constraints also leads to precise values for the corresponding multipliers which is the situation discussed in this paper. To induce fluctuations in charge and mass, one must require that the corresponding constraints be imprecisely specified. The discussion of the physical conditions that might possibly bring about such interesting effects lies beyond the scope of the present study.

So far we have discussed ED on the larger unconstrained e-phase space $T^{*}S^{+}$. Next we discuss the additional requirements that implement gauge invariance and yield proper entropic electrodynamics.

## 6. Quantum Electrodynamics

### 6.1. Gauge Invariance and the Gauss Constraint

We require that probabilities, phases, and wave functions reflect the consequences of invariance under the local gauge transformation.

(75) $$A_{ax}\to A_{ax}^{\xi }=A_{ax}+\partial \xi_{x}/\partial x^{a}\;.$$

The main constraint follows from the observation that *A* and $A^{\xi }$ represent the same ontic microstate, and therefore, the corresponding probabilities must be equal.

(76) $$\rho \left[z,A^{\xi }\right]\approx \rho \left[z,A\right]\;.$$

(We adopt Dirac’s weak equality ≈ notation that holds once the constraints are implemented.) This can be expressed in differential form: Taylor-expanding for small ξ, we obtain the identity

(77) $$\rho_{z,A+\partial \xi }=\rho_{zA}+\int d^{3}x\;\frac{\delta \rho_{zA}}{\delta A_{ax}}\partial_{ax}\xi_{x}\approx \rho_{zA}\;,$$

which must hold for arbitrary choices of $\xi_{x}$. Therefore, the allowed ρs are constrained to satisfy

(78) $$\partial_{ax}\frac{\delta \rho_{zA}}{\delta A_{ax}}\approx 0\;.$$

Analogous conditions apply to any gauge-invariant functional.

The invariance of $\rho_{zA}$ must be preserved by time evolution, and this determines the gauge transformation of the drift potential $\varphi_{zA}$ and the phase $\phi_{zA}$. From Equations (52)–(54) we see that ρ remains invariant under $A_{ax}\to A_{ax}^{\xi }$ provided $\varphi_{zA}$ and $\phi_{zA}$ transform according to the local gauge transformations,

(79) $$\begin{array}{cc} \varphi_{zA} & \to \varphi_{zA}^{\xi }\approx \varphi_{zA}+\sum_{n}\frac{q_{n}}{c}\xi_{z_{n}}\;, \end{array}$$

(80) $$\begin{array}{cc} \phi_{zA} & \to \phi_{zA}^{\xi }\approx \phi_{zA}+\sum_{n}\frac{q_{n}}{c}\xi_{z_{n}}\;, \end{array}$$

where $\xi_{x}$ is a function on the 3d-space X. The dynamical preservation of the gauge symmetry follows from the fact that the original constraints (36) and (37) are not independent of each other—both are linear in the same displacements $\langle \Delta z_{n}^{a}\rangle$—which leads to the invariant combination $\left(\partial_{na}\phi_{zA}-q_{n}A_{az_{n}}\right)$ in (53). Thus, our particular choice of entropic prior and constraints has led to an ED that allows a gauge-covariant HK flow.

We require that $\phi_{zA}$ transforms according to (80), but being a functional of *A*, we can also calculate $\phi_{zA}^{\xi }$ directly:

(81) $$\phi_{zA}^{\xi }=\phi_{zA^{\xi }}\;\mathrm{with}\;\phi_{zA^{\xi }}=\phi_{zA}+\int d^{3}x\;\frac{\delta \phi_{zA}}{\delta A_{ax}}\partial_{ax}\xi_{x}\;.$$

Therefore, equating (81) to (80), the change in $\phi_{zA}$ is

(82) $$\delta_{\xi }\phi_{zA}=\int d^{3}x\;\frac{\delta \phi_{zA}}{\delta A_{ax}}\partial_{ax}\xi_{x}\approx \sum_{n}\frac{q_{n}}{c}\xi_{z_{n}}\;,$$

which, after integrating by parts and rearranging, gives

(83) $$\int d^{3}x\;\left(\partial_{ax}\frac{\delta \phi_{zA}}{\delta A_{ax}}+\sum_{n}\frac{q_{n}}{c}\delta_{z_{n}x}\right)\xi_{x}\approx 0\;.$$

Since this identity must hold for arbitrary choices of $\xi_{x}$, the allowed phases are constrained to satisfy the infinite set of constraints (one at every point x∈X),

(84) $$\partial_{ax}\frac{\delta \phi_{zA}}{\delta A_{ax}}+\sum_{n}\frac{q_{n}}{c}\delta_{z_{n}x}\approx 0\;.$$

Having established the constraints on ρs and ϕs, we can find the constraints on wave functions $\psi =\rho^{1/2}e^{i\phi /\hbar }$. Using (76) and (80) and Taylor-expanding gives

(85) $$\psi_{zA}^{\xi }\approx \psi_{zA}\exp\left(\frac{i}{\hbar }\sum_{n}\frac{q_{n}}{c}\xi_{z_{n}}\right)=\psi_{zA}\left(1+\frac{i}{\hbar }\sum_{n}\frac{q_{n}}{c}\xi_{z_{n}}\right)\;.$$

But we can also calculate $\psi_{zA}^{\xi }$ directly:

(86) $$\psi_{zA}^{\xi }=\psi_{z,A+\partial \xi }=\psi_{zA}+\int d^{3}x\;\frac{\delta \psi_{zA}}{\delta A_{ax}}\partial_{ax}\xi_{x}\;.$$

Therefore,

(87) $$\delta_{\xi }\psi_{zA}=\int d^{3}x\;\frac{\delta \psi_{zA}}{\delta A_{ax}}\partial_{ax}\xi_{x}\approx \psi_{zA}\frac{i}{\hbar c}\sum_{n}q_{n}\xi_{z_{n}}\;,$$

which, after some algebra, leads to

(88) $$\left[\int d^{3}x\;\xi_{x}\left(\partial_{ax}\frac{\hbar c}{i}\frac{\delta }{\delta A_{ax}}+\sum_{n}q_{n}\delta_{z_{n}x}\right)\right]\psi_{zA}\approx 0\;.$$

Once again, since $\xi_{x}$ is an arbitrary function, we conclude that the requirement of gauge invariance restricts the allowed quantum states ψ to those that satisfy the infinite set of constraints (one at every point x∈X)

(89) $$\left(\partial_{ax}i\hbar c\frac{\delta }{\delta A_{ax}}-\sum_{n}q_{n}\delta_{z_{n}x}\right)\psi_{zA}\approx 0\;,$$

which is recognized as the quantum version of Gauss’ law,

(90) $$\left(\partial_{ax}{\hat{E}}_{x}^{a}-{\hat{\rho }}_{x}^{e}\right)\psi_{zA}\approx 0\;,$$

provided one identifies

(91) $${\hat{E}}^{a}\left(x\right)=i\hbar c\frac{\delta }{\delta A_{a}\left(x\right)}$$

as the representation of the electric field operator ${\hat{E}}^{a}\left(x\right)$ acting on functionals ψ[z,A] of $A_{a}\left(x\right)$ and ${\hat{\rho }}_{x}^{e}$ is the electric charge density, Equation (69). In classical electrodynamics Equation (91) corresponds to the assertion that the electric field (times −1/c) is the momentum that is canonically conjugate to the potential $A_{a}\left(x\right)$.

Incidentally, the derivative operator ${\hat{E}}^{a}$ is how electric fields are introduced in the ED approach. ED is founded on the recognition that the ontic degrees of freedom are the radiation field, which is redundantly represented by the vector potential $A_{ax}$. And that is all there is; there is no such thing as an *ontic* electric field!

### 6.2. Time Evolution

From now on we adopt the Hamiltonian $\hat{H}$ given by Equation (65) and, to be specific, the potential ${\hat{V}}_{zA}$ is given in Equation (67):

(92) $$\hat{H}={\hat{H}}^{\mathrm{mat}}+{\hat{H}}^{\mathrm{rad}}\;,$$

where

(93) $${\hat{H}}^{\mathrm{mat}}=\sum_{n}\frac{1}{2m_{n}}\delta^{ab}\frac{\hbar }{i}D_{na}\frac{\hbar }{i}D_{nb}\;\mathrm{and}\;{\hat{H}}^{\mathrm{rad}}=\frac{1}{2}\int d^{3}x\;\left({\hat{E}}_{x}^{2}+{\hat{B}}_{x}^{2}\right)\;.$$

The mere existence of the Gauss constraint, Equation (90), induces an additional change. As pointed out by Dirac [8], it leads us to consider the modified Hamiltonian,

(94) $${\hat{H}}_{\Phi }=\hat{H}+{\hat{\Gamma }}_{\Phi }\;\mathrm{with}\;{\hat{\Gamma }}_{\Phi }=\int d^{3}x\;\Phi_{xt}\left({\hat{\rho }}_{x}^{e}-\partial_{ax}{\hat{E}}_{x}^{a}\right)$$

where $\Phi_{xt}=\Phi_{xt}\left[z_{n},A\right]$ is some well-behaved but otherwise arbitrary function of *x*, *t*, and of the ontic variables $z_{n}$ and $A_{x}^{a}$.

**Remark** **1.** *In the Dirac approach to the* classical *dynamics of constrained Hamiltonian systems [8], there is one quantity* Φ *for each constraint, which accounts for $\Phi =\Phi_{xt}$ being a function of x, and at each x the quantity $\Phi_{xt}$ is an arbitrary functional of the canonical variables $z_{n}$ and $A_{x}^{a}$ and* their *respective momenta. In the ED approach to* quantum dynamics there is an important difference: the canonical variables are ψ and $\psi^{*}$, not $z_{n}$, $A_{x}^{a}$, and their momenta. The HK flows require that the Hamiltonians $\tilde{H}$ and ${\tilde{H}}_{\Phi }$ be bilinear in ψ and $\psi^{*}$, Equation (64), which in turn implies that the kernels $\hat{H}$ and ${\hat{H}}_{\Phi }$ must be independent of the canonical variables ψ and $\psi^{*}$. Thus ${\hat{\Gamma }}_{\Phi }$ can depend on $z_{n}$ and $A_{x}^{a}$ but not on ψ and $\psi^{*}$.

The new Schrödinger equation is

(95) $$i\hbar \frac{\partial \psi }{\partial t}={\hat{H}}_{\Phi }\psi =\left(\hat{H}+{\hat{\Gamma }}_{\Phi }\right)\psi \;.$$

Imposing ${\hat{\Gamma }}_{\Phi }\psi \approx 0$ shows that the arbitrary function $\Phi_{x}$ has no effect on the evolution of ψ, but it does introduce indeterminism in the evolution of gauge-dependent fields such as ${\tilde{A}}_{ax}$ (see Equation (136)).

The consistency of the whole scheme requires that the Gauss constraint be preserved by time evolution, that is,

(96) $$\mathrm{if}\;{\hat{\Gamma }}_{\Phi }\psi \approx 0\;\mathrm{then}\;\partial_{t}\left({\hat{\Gamma }}_{\Phi }\psi \right)\approx 0\;.$$

More explicitly,

$$\begin{array}{cc} \partial_{t}\left({\hat{\Gamma }}_{\Phi }\psi \right) & =\int d^{3}x\;{\dot{\Phi }}_{xt}\left({\hat{\rho }}_{x}^{e}-\partial_{ax}{\hat{E}}_{x}^{a}\right)\psi +\frac{1}{i\hbar }\int d^{3}x\;\Phi_{xt}\left({\hat{\rho }}_{x}^{e}-\partial_{ax}{\hat{E}}_{x}^{a}\right){\hat{H}}_{\Phi }\psi \\ \; & \approx \frac{1}{i\hbar }\int d^{3}x\;\Phi_{xt}\left(\hat{H}\left({\hat{\rho }}_{x}^{e}-\partial_{ax}{\hat{E}}_{x}^{a}\right)\psi +\left[{\hat{\rho }}_{x}^{e}-\partial_{ax}{\hat{E}}_{x}^{a},\hat{H}\right]\psi \right)\;, \end{array}$$

so that

(97) $$\partial_{t}\left({\hat{\Gamma }}_{\Phi }\psi \right)\approx \frac{1}{i\hbar }\int d^{3}x\;\Phi_{xt}\left[\left({\hat{\rho }}_{x}^{e}-\partial_{ax}{\hat{E}}_{x}^{a}\right),\hat{H}\right]\psi \;,$$

which requires that we evaluate the commutator.

First we consider the commutators $\left[{\hat{\rho }}_{x}^{e},{\hat{H}}^{\mathrm{rad}}\right]$ and $\left[\partial_{ax}{\hat{E}}_{x}^{a},{\hat{H}}^{\mathrm{rad}}\right]$. The former vanishes trivially. For the latter we need $\left[{\hat{E}}_{x}^{a},{\hat{B}}_{x^{\prime }}^{b}\right]$, which is

(98) $$\left[{\hat{E}}_{x}^{a},{\hat{B}}_{x^{\prime }}^{b}\right]\psi_{zA}=i\hbar c\varepsilon^{bcd}\partial_{cx^{\prime }}\left[\frac{\delta }{\delta A_{ax}},A_{dx^{\prime }}\right]\psi_{zA}=i\hbar c\varepsilon^{abc}\left(\partial_{cx^{\prime }}\delta_{x^{\prime }x}\right)\psi_{zA}\;,$$

and implies that

(99) $$\left[{\hat{E}}_{x}^{a},{\hat{H}}^{\mathrm{rad}}\right]\psi_{zA}=\frac{1}{2}\int d^{3}x\;\left[{\hat{E}}_{x}^{a},{\hat{B}}_{x}^{2}\right]\psi_{zA}=i\hbar c{\left(\partial_{x}\times {\hat{B}}_{x}\right)}^{a}\psi_{zA}\;,$$

and

(100) $$\left[\partial_{ax}{\hat{E}}_{x}^{a},{\hat{H}}^{\mathrm{rad}}\right]\psi_{zA}=i\hbar c\partial_{ax}{\left(\partial_{x}\times {\hat{B}}_{x}\right)}^{a}\psi_{zA}=0\;,$$

Therefore,

(101) $$\left[{\hat{\rho }}_{x}^{e}-\partial_{ax}{\hat{E}}_{x}^{a},{\hat{H}}^{\mathrm{rad}}\right]\psi_{zA}=0\;.$$

Next we deal with $\left[\partial_{ax}{\hat{E}}_{x}^{a},{\hat{H}}^{\mathrm{mat}}\right]$ and $\left[{\hat{\rho }}_{x}^{e},{\hat{H}}^{\mathrm{mat}}\right]$. For the former, we use

(102) $$\left[{\hat{E}}_{x}^{a},D_{nb}\right]\psi_{zA}=q_{n}\delta_{b}^{a}\delta_{xz_{n}}\psi_{zA}\;,$$

and

(103) $$\delta^{bc}\left[{\hat{E}}_{x}^{a},D_{nb}D_{nc}\right]\psi_{zA}=2q_{n}\delta^{ab}\delta_{xz_{n}}D_{nb}\psi_{zA}+q_{n}\delta^{ab}\frac{\partial \delta_{xz_{n}}}{\partial z_{n}^{b}}\psi_{zA}\;,$$

which, substituting ${\hat{H}}^{\mathrm{mat}}$ from (93), implies that

(104) $$\partial_{ax}\left[{\hat{E}}_{x}^{a},{\hat{H}}^{\mathrm{mat}}\right]\psi_{zA}=\sum_{n}\frac{-\hbar^{2}q_{n}}{m_{n}}\left(\delta^{ab}\partial_{ax}\delta_{xz_{n}}D_{nb}-\frac{1}{2}\partial_{x}^{2}\delta_{xz_{n}}\right)\psi_{zA}\;.$$

Next consider $\left[{\hat{\rho }}_{x}^{e},{\hat{H}}^{\mathrm{mat}}\right]$. We use (69) and (58), so that

(105) $$\left[{\hat{\rho }}_{x}^{e},D_{na}\right]\psi =\sum_{n^{\prime }}q_{n^{\prime }}\left[\delta_{z_{n^{\prime }}x},\frac{\partial }{\partial z_{n}^{a}}-\frac{iq_{n}}{\hbar c}A_{az_{n}}\right]\psi =q_{n}\frac{\partial \delta_{z_{n}x}}{\partial x^{a}}\psi \;,$$

and

(106) $$\begin{array}{cc} \delta^{ab}\left[{\hat{\rho }}_{x}^{e},D_{na}D_{nb}\right]\psi & =\delta^{ab}\left[{\hat{\rho }}_{x}^{e},D_{na}\right]D_{nb}\psi +\delta^{ab}D_{na}\left[{\hat{\rho }}_{x}^{e},D_{nb}\right]\psi \end{array}$$

(107) $$\begin{array}{cc} \; & =2q_{n}\delta^{ab}\frac{\partial \delta_{z_{n}x}}{\partial x^{a}}D_{nb}\psi -q_{n}\partial_{x}^{2}\delta_{xz_{n}}\psi \;. \end{array}$$

Therefore, substituting ${\hat{H}}^{\mathrm{mat}}$ from (93),

(108) $$\left[{\hat{\rho }}_{x}^{e},{\hat{H}}^{\mathrm{mat}}\right]\psi =\sum_{n}\frac{-\hbar^{2}q_{n}}{m_{n}}\left(\delta^{ab}\frac{\partial \delta_{z_{n}x}}{\partial z_{n}^{a}}D_{nb}-\frac{1}{2}\partial_{x}^{2}\delta_{xz_{n}}\right)\psi \;.$$

Combining (104) and (108) leads to

(109) $$\left[{\hat{\rho }}_{x}^{e}-\partial_{ax}{\hat{E}}_{x}^{a},{\hat{H}}^{\mathrm{mat}}\right]\psi_{zA}=0\;,$$

which, together with (101), implies that

(110) $$[\left({\hat{\rho }}_{x}^{e}-\partial_{ax}{\hat{E}}_{x}^{a}\right),\hat{H}]=0\;,$$

and proves (97). Note that (110) is not a weak equality; it holds strongly.

We can also check the mutual consistency of time evolution and time-dependent gauge transformations. Write Equation (85) as $\psi^{\xi }={\hat{U}}_{\xi }\psi$, where ${\hat{U}}_{\xi }$ is the unitary operator

(111) $${\hat{U}}_{\xi }=\exp\left(\frac{i}{\hbar c}\sum_{n}q_{n}\xi_{z_{n}}\right)=\exp\left(\frac{i}{\hbar c}\int d^{3}x\;\xi_{x}{\hat{\rho }}_{x}^{e}\right)\;.$$

Then, the consistency of (95) with the transformed Schrödinger equation,

(112) $$i\hbar \frac{\partial \psi^{\xi }}{\partial t}={\hat{H}}_{\Phi }^{\xi }\psi^{\xi }\;,$$

requires that

(113) $$i\hbar \frac{\partial }{\partial t}\left({\hat{U}}_{\xi }\psi \right)=i\hbar \frac{\partial {\hat{U}}_{\xi }}{\partial t}\psi +{\hat{U}}_{\xi }H_{\Phi }\psi ={\hat{H}}_{\Phi }^{\xi }{\hat{U}}_{\xi }\psi$$

or

(114) $${\hat{H}}_{\Phi }^{\xi }={\hat{U}}_{\xi }H_{\Phi }{\hat{U}}_{\xi }^{-1}+i\hbar \frac{\partial {\hat{U}}_{\xi }}{\partial t}{\hat{U}}_{\xi }^{-1}\;.$$

Substituting (111), we find

(115) $${\hat{H}}_{\Phi }^{\xi }={\hat{H}}_{\Phi }+\delta_{\xi }\hat{V}\;\mathrm{with}\;\delta_{\xi }\hat{V}=-\frac{1}{c}\int d^{3}x\;\partial_{t}\xi_{x}{\hat{\rho }}_{x}^{e}\;.$$

Thus, we discover the familiar fact that the Hamiltonian is not invariant under time-dependent gauge transformations and that the effect of the latter is to cause a shift in the background scalar potential.

### 6.3. Gauge Transformations as HK Flows

Consider the Hamiltonian functional that describes the expected electric charge density smeared by an arbitrary function $\zeta_{x}$,

(116) $$\begin{array}{cc} {\tilde{Q}}_{\zeta }^{e} & =\int d^{3}x\;\zeta_{x}\left(\int dzDA\;\rho_{zA}{\hat{\rho }}_{x}^{e}\right) \end{array}$$

(117) $$\begin{array}{cc} \; & =\int dzDA\;\psi_{zA}^{*}{\hat{Q}}_{\zeta }^{e}\psi_{zA}\;\mathrm{with}\;{\hat{Q}}_{\zeta }^{e}=\int d^{3}x\;\zeta_{x}{\hat{\rho }}_{x}^{e}\;. \end{array}$$

It generates an HK flow $\psi_{zA}\left(\lambda \right)$ parametrized by λ and defined by the Hamilton/Schrödinger equation,

(118) $$\frac{\partial \psi_{zA}}{\partial \lambda }=\left\{\psi_{zA},{\tilde{Q}}_{\zeta }^{e}\right\}=\frac{1}{i\hbar }\frac{\delta {\tilde{Q}}_{\zeta }^{e}}{\delta \psi_{zA}^{*}}=\frac{1}{i\hbar }{\hat{Q}}_{\zeta }^{e}\psi_{zA}\;.$$

The corresponding integral curve is

(119) $$\psi_{zA}\left(\lambda \right)=\psi_{zA}\left(0\right)\exp\left(\frac{\lambda }{i\hbar }{\hat{Q}}_{\zeta }^{e}\right)=\psi_{zA}\left(0\right)\exp\left(\frac{\lambda }{i\hbar }\int d^{3}x\;\zeta_{x}{\hat{\rho }}_{x}^{e}\right)\;.$$

Compared with (85), this is recognized as the gauge orbit generated by gauge transformations with function $\xi_{x}=-c\lambda \zeta_{x}$. In other words, the smeared expected charge density ${\tilde{Q}}_{\zeta }^{e}$ is the generator of gauge transformations.

We can also consider the Hamiltonian function that describes the smeared Gauss constraint, Equation (94),

(120) $${\tilde{\Gamma }}_{\Phi }=\int dzDA\;\psi_{zA}^{*}{\hat{\Gamma }}_{\Phi }\psi_{zA}\;\mathrm{with}\;{\hat{\Gamma }}_{\Phi }=\int d^{3}x\;\Phi_{xt}\left({\hat{\rho }}_{x}^{e}-\partial_{ax}{\hat{E}}_{x}^{a}\right)\;.$$

The corresponding HK flow $\psi_{zA}\left(\lambda \right)$ is given by

(121) $$\frac{\partial \psi_{zA}}{\partial \lambda }=\left\{\psi_{zA},{\tilde{\Gamma }}_{\Phi }\right\}=\frac{1}{i\hbar }\frac{\delta {\tilde{\Gamma }}_{\Phi }}{\delta \psi_{zA}^{*}}=\frac{1}{i\hbar }{\tilde{\Gamma }}_{\Phi }\psi_{zA}\;$$

and its integral curves are

(122) $$\psi_{zA}\left(\lambda \right)=\exp\left(\frac{\lambda }{i\hbar }{\tilde{\Gamma }}_{\Phi }\right)\psi_{zA}\left(0\right)\;.$$

We see that if ${\hat{\Gamma }}_{\Phi }\psi_{zA}\left(0\right)\approx 0$, then ${\hat{\Gamma }}_{\Phi }\psi_{zA}\left(\lambda \right)\approx 0$ too, that is, ${\tilde{\Gamma }}_{\Phi }$ generate curves along which the Gauss constraint is obeyed.

**Remark** **2.** *According to Dirac’s hypothesis, constraints that commute with all other constraints (called first-class constraints) are the generators of gauge transformations [8]. Even though Dirac’s hypothesis holds true for those cases he tested, in the context of ED the Dirac hypothesis fails. Indeed, we can easily check that the commutators*

(123) $$[{\hat{\Gamma }}_{\Phi },{\hat{\Gamma }}_{\Phi^{\prime }}]=0\;$$

*vanish strongly so that the constraints ${\hat{\Gamma }}_{\Phi }$ are first class, but Equation (122) clearly shows that ${\hat{\Gamma }}_{\Phi }$ does not generate gauge transformations.*

### 6.4. Maxwell Equations

We have just concluded our formulation of the ED approach to non-relativistic quantum electrodynamics. In the next two sections we shall offer some further evidence that despite ED’s unorthodox foundations, which include clarity about which variables play an ontic and which an epistemic role and the absence of ontic dynamics, the ED model developed here is empirically equivalent to the standard versions of QED.

We saw, in Equations (67) and (91), that the electric $E_{x}^{a}$ and the magnetic $B_{x}^{a}$ fields are not fundamental; they are derivative, auxiliary concepts related to the concept that is actually fundamental, which is the vector potential $A_{x}^{a}$. Let

(124) $$\begin{array}{cc} {\tilde{A}}_{x}^{a} & =\int dzDA\;\psi_{zA}^{*}{\hat{A}}_{x}^{a}\psi_{zA}\;\mathrm{with}\;{\hat{A}}_{x}^{a}\overset{\mathrm{def}}{=}A_{x}^{a}\;, \end{array}$$

(125) $$\begin{array}{cc} {\tilde{B}}_{x}^{a} & =\int dzDA\;\psi_{zA}^{*}{\hat{B}}_{x}^{a}\psi_{zA}\;\mathrm{with}\;{\hat{B}}_{x}^{a}\overset{\mathrm{def}}{=}{\left(\partial \times \hat{A}\right)}_{x}^{a}\;, \end{array}$$

(126) $$\begin{array}{cc} {\tilde{E}}_{x}^{a} & =\int dzDA\;\psi_{zA}^{*}{\hat{E}}_{x}^{a}\psi_{zA}\;\mathrm{with}\;{\hat{E}}_{x}^{a}\overset{\mathrm{def}}{=}i\hbar c\frac{\delta }{\delta A_{ax}}\;. \end{array}$$

Two of the Maxwell equations we have already discussed. The absence of magnetic monopoles follows from the definition of the ${\hat{B}}_{x}$ field, Equation (67):

(127) $${\hat{B}}_{x}\overset{\mathrm{def}}{=}\partial_{x}\times {\hat{A}}_{x}\Rightarrow \tilde{B}=\partial_{x}\times {\tilde{A}}_{x}\Rightarrow \partial_{x}\cdot {\tilde{B}}_{x}=0\;.$$

Gauss’ law is the Gauss constraint (Equation (90)), to be imposed on wave functions:

(128) $$\left(\partial_{ax}{\hat{E}}_{x}^{a}-{\hat{\rho }}_{x}^{e}\right)\psi_{zA}\approx 0\;\mathrm{or}\;\partial_{ax}{\tilde{E}}_{x}^{a}\approx {\tilde{\rho }}_{x}^{e}\;.$$

The other two Maxwell equations are equations of time evolution for ${\tilde{A}}_{x}^{a}$, ${\tilde{B}}_{x}^{a}$, and ${\tilde{E}}_{x}^{a}$,

(129) $$\partial_{t}{\tilde{A}}_{x}^{a}=\left\{{\tilde{A}}_{x}^{a},{\tilde{H}}_{\Phi }\right\}\;\mathrm{and}\;\partial_{t}{\tilde{E}}_{x}=\left\{{\tilde{E}}_{x}^{a},{\tilde{H}}_{\Phi }\right\}\;.$$

We use (93) and (94) to write the modified Hamiltonian as

(130) $${\hat{H}}_{\Phi }={\hat{H}}_{\Phi }^{\mathrm{mat}}+{\hat{H}}_{\Phi }^{\mathrm{rad}}\;,$$

where

(131) $${\hat{H}}_{\Phi }^{\mathrm{mat}}={\hat{H}}^{\mathrm{mat}}+\int d^{3}x\;\Phi_{x}{\hat{\rho }}_{x}^{e}\;\mathrm{and}\;{\hat{H}}_{\Phi }^{\mathrm{rad}}={\hat{H}}^{\mathrm{rad}}+\int d^{3}x\;{\hat{E}}_{x}^{a}\partial_{ax}\Phi_{x}$$

where the last term has been integrated by parts. Calculating the PBs leads to

(132) $$\begin{array}{cc} \partial_{t}{\tilde{A}}_{x}^{a} & =\frac{1}{i\hbar }\int dzDA\;\psi_{zA}^{*}\left[A_{x}^{a},{\hat{H}}_{\Phi }\right]\psi_{zA}\;, \end{array}$$

(133) $$\begin{array}{cc} \partial_{t}{\tilde{E}}_{x}^{a} & =\frac{1}{i\hbar }\int dzDA\;\psi_{zA}^{*}\left[{\hat{E}}_{x}^{a},{\hat{H}}_{\Phi }\right]\psi_{zA}\;. \end{array}$$

The *A*-commutators give

(134) $$\left[A_{x}^{a},{\hat{H}}_{\Phi }^{\mathrm{mat}}\right]\psi_{zA}=0\;\mathrm{and}\;\left[A_{x}^{a},{\hat{H}}_{\Phi }^{\mathrm{rad}}\right]\psi_{zA}=-i\hbar c\;\left({\hat{E}}_{x}^{a}+\partial_{x}^{a}\Phi_{x}\right)\psi_{zA}\;.$$

The first equation is trivial; the second only slightly less so. We use

(135) $$\left[A_{x}^{a},{\hat{E}}_{x^{\prime }}^{b}\right]\psi =i\hbar c\left[A_{x}^{a},\frac{\delta }{\delta A_{bx^{\prime }}}\right]\psi =-i\hbar c\delta^{ab}\delta_{xx^{\prime }}\;,$$

and (134) follows.

Substituting this into (132), the time evolution of $\tilde{A}$ is

(136) $$\partial_{t}{\tilde{A}}_{x}^{a}=-c\left({\tilde{E}}_{x}^{a}+\partial_{x}^{a}\Phi_{x}\right)\;.$$

This shows that the longitudinal component of $\partial_{t}{\tilde{A}}^{a}$ is quite arbitrary, which is the defining characteristic of gauge fields. We can make this explicit by decomposing $A_{x}^{a}$ into its longitudinal and transverse parts,

(137) $$A_{x}^{a}=A_{\ell x}^{a}+A_{\mathrm{tr}x}^{a}\;,$$

with

(138) $$\partial_{x}\times A_{\ell x}=0\;\mathrm{and}\;\partial_{x}\cdot A_{\mathrm{tr}x}=0\;,$$

so that $A_{\ell }^{a}$ is the gauge-dependent component and $A_{\mathrm{tr}}^{a}$ is gauge-invariant. We can also write (136) as

(139) $${\tilde{E}}_{x}^{a}=-\partial_{x}^{a}\Phi_{x}-\frac{1}{c}\partial_{t}{\tilde{A}}_{x}^{a}$$

which, if we interpret the function $\Phi_{xt}$ as a scalar potential, recovers the familiar expression for the electric field in terms of the potentials $\Phi_{x}$ and ${\tilde{A}}_{x}^{a}$. At this point we can *extend* the gauge transformations to the scalar potential $\Phi_{xt}$, and write

(140) $${\tilde{A}}_{\mathrm{tr}x}^{\xi }={\tilde{A}}_{\mathrm{tr}x}\;,\;{\tilde{A}}_{\ell x}^{\xi }={\tilde{A}}_{\ell x}+\partial_{x}\xi_{x}\;\mathrm{and}\;\Phi_{x}^{\xi }=\Phi_{x}-\frac{1}{c}\xi_{x}\;,$$

which makes the electric field (139) gauge invariant. This is a shift in perspective that warrants emphasis: In standard approaches to electrodynamics, whether classical or quantum, an identity such as (139) is taken as part of the definition of the potentials from the already given, presumably fundamental, classical field strengths. In the ED approach, (139) is a derived result. The fundamental equation is (136); it describes the time evolution of the field ${\tilde{A}}_{x}^{a}$ in terms of the quantities ${\tilde{E}}_{x}^{a}$ and $\Phi_{x}$ introduced by the Gauss constraint, Equations (90)–(94).

The third Maxwell equation follows immediately; we just take the curl of (139) to find Faraday’s law,

(141) $$\partial_{x}\times {\tilde{E}}_{x}=-\frac{1}{c}\partial_{t}{\tilde{B}}_{x}\;.$$

Next we deal with (133). Since

(142) $$\begin{array}{cc} \; & \left[{\hat{E}}_{x}^{a},{\hat{\Gamma }}_{\Phi }\right]\psi =i\hbar c\int d^{3}x\;\left[\frac{\delta }{\delta A_{ax}},\;\Phi_{x}\left({\hat{\rho }}_{x}^{e}-\partial_{ax}{\hat{E}}_{x}^{a}\right)\right]\psi \\ \; & =i\hbar c\int d^{3}x\;\left(\Phi_{x}\left[\frac{\delta }{\delta A_{ax}},\;\left({\hat{\rho }}_{x}^{e}-\partial_{ax}{\hat{E}}_{x}^{a}\right)\right]+\left[\frac{\delta }{\delta A_{ax}},\;\Phi_{x}\right]\left({\hat{\rho }}_{x}^{e}-\partial_{ax}{\hat{E}}_{x}^{a}\right)\right)\psi \approx 0\;, \end{array}$$

we can replace $\left({\hat{H}}_{\Phi }^{\mathrm{rad}},{\hat{H}}_{\Phi }^{\mathrm{mat}}\right)$ with $\left({\hat{H}}^{\mathrm{rad}},{\hat{H}}^{\mathrm{mat}}\right)$ in the commutators. The commutator $\left[{\hat{E}}_{x}^{a},{\hat{H}}^{\mathrm{rad}}\right]$ is given in (99). The commutator $\left[{\hat{E}}_{x}^{a},{\hat{H}}^{\mathrm{mat}}\right]$ requires more algebra; we use (103) and substitute it into

(143) $$\frac{1}{i\hbar }\int dzDA\;\psi^{*}\left[{\hat{E}}_{x}^{a},{\hat{H}}^{\mathrm{mat}}\right]\psi =\int dzDA\;\sum_{n}\frac{i\hbar q_{n}}{m_{n}}\delta^{ab}\delta_{xz_{n}}\left(\psi^{*}D_{nb}\psi -\frac{1}{2}\frac{\partial \rho_{zA}}{\partial z_{n}^{b}}\right)\;.$$

This can be simplified considerably. From $\psi =\rho^{1/2}e^{i\phi /\hbar }$, we obtain the identity

(144) $$\psi_{zA}^{*}\frac{\partial }{\partial z_{n}^{b}}\psi_{zA}-\frac{1}{2}\frac{\partial \rho_{zA}}{\partial z_{n}^{b}}=\frac{i}{\hbar }\psi_{zA}^{*}\frac{\partial \phi_{zA}}{\partial z_{n}^{b}}\psi_{zA}\;,$$

which, when combined with Equations (53) and (71), leads to

(145) $$\frac{1}{i\hbar }\int dzDA\;\psi_{zA}^{*}\left[{\hat{E}}_{x}^{a},{\hat{H}}^{\mathrm{mat}}\right]\psi_{zA}=\int dzDA\;\rho_{zA}\sum_{n}q_{n}\delta_{z_{n}x}v_{n}^{a}=-{\tilde{J}}_{x}^{ea}\;.$$

Finally, substitute (99) and (145) into (133) to find the fourth Maxwell equation, the Ampère-Maxwell law,

(146) $$\partial_{t}{\tilde{E}}_{x}=c\partial_{x}\times {\tilde{B}}_{x}-{\tilde{J}}_{x}^{e}\;.$$

This derivation of the Maxwell equations has taken full account of the redundancy of the representation of the $A_{x}^{a}$ field, but it has been carried out without having specified a gauge condition. In other words, the derivation holds for all gauges. However, in applications, there is no reason not take advantage of the freedom to choose a convenient gauge; this we do next.

### 6.5. The Coulomb Potential

The Gauss constraint is a restriction on the longitudinal component of the electric field operator. This suggests decomposing the vector operator ${\hat{E}}_{x}$ into its longitudinal and transverse parts,

(147) $$\hat{E}={\hat{E}}_{\ell }+{\hat{E}}_{\mathrm{tr}}\;\mathrm{with}\;\partial_{x}\times {\hat{E}}_{\ell x}=0\;\mathrm{and}\;\partial_{x}\cdot {\hat{E}}_{\mathrm{tr}x}=0$$

where, using (139), we obtain

(148) $${\tilde{E}}_{\ell x}^{a}=-\partial_{x}^{a}\Phi_{x}-\frac{1}{c}\partial_{t}{\tilde{A}}_{\ell x}^{a}\;.$$

We can take advantage of (140) to impose the Coulomb gauge,

(149) $${\tilde{A}}_{\ell x}^{a}=0\;\mathrm{or}\;\partial_{a}{\tilde{A}}_{x}^{a}=0\;.$$

Then ${\hat{E}}_{\ell \;}$ can be written as the gradient of a scalar potential operator ${\hat{\Phi }}_{x}^{C}$,

(150) $${\hat{E}}_{\ell x}=-\partial_{x}{\hat{\Phi }}_{x}^{C}\;,\;{\hat{E}}_{\mathrm{tr}x}={\hat{E}}_{x}+\partial_{x}{\hat{\Phi }}_{x}^{C}\;,$$

and the Gauss constraint reads

(151) $$\left(\partial_{a}{\hat{E}}_{\ell }^{a}-{\hat{\rho }}^{e}\right)\psi \approx 0\;\mathrm{or}\;\left(\partial_{a}\partial^{a}{\hat{\Phi }}^{C}+{\hat{\rho }}^{e}\right)\psi \approx 0\;,$$

which is the quantum version of the Poisson equation. The solution to (151) is the Coulomb potential,

(152) $${\hat{\Phi }}_{x}^{C}\psi \approx \int d^{3}x^{\prime }\frac{{\hat{\rho }}_{x^{\prime }}^{e}}{4\pi |\vec{x}-{\vec{x}}^{\prime }|}\psi =\sum_{n}\frac{q_{n}}{4\pi |\vec{x}-{\vec{z}}_{n}|}\psi \;,$$

where we used (69). This reproduces a well-known result in QED, namely, that the scalar potential ${\hat{\Phi }}_{x}^{C}\left(z\right)$ is not an independent dynamical variable but a function of the particle positions. (Note that $\Phi_{x}^{C}$ is not a function of the canonical variables ψ and $\psi^{*}$.)

In terms of ${\hat{E}}_{\ell x}$ and ${\hat{E}}_{\mathrm{tr}x}$, and imposing the Gauss constraint, the Hamiltonian (130) now reads

(153) $$\hat{H}\approx \sum_{n}\frac{-\hbar^{2}}{2m_{n}}\delta^{ab}D_{na}D_{nb}+\int d^{3}x\;\frac{1}{2}\delta_{ab}\left(\partial^{a}{\hat{\Phi }}^{C}\partial^{b}{\hat{\Phi }}^{C}+{\hat{E}}_{\mathrm{tr}}^{a}{\hat{E}}_{\mathrm{tr}}^{b}+{\hat{B}}^{a}{\hat{B}}^{b}\right)\;.$$

Integrating the Coulomb term by parts and using the Poisson equation yields the familiar result

(154) $$\int d^{3}x\;\frac{1}{2}\delta_{ab}\partial^{a}{\hat{\Phi }}^{C}\partial^{b}{\hat{\Phi }}^{C}=\frac{1}{2}\int d^{3}x\;{\hat{\Phi }}^{C}{\hat{\rho }}^{e}=\frac{1}{2}\sum_{n\neq n^{\prime }}\frac{q_{n}q_{n^{\prime }}}{4\pi |{\vec{z}}_{n}-{\vec{z}}_{n^{\prime }}|}\;,$$

where we have invoked the usual “argument” that the infinite constant contributed by the self-energy terms $n=n^{\prime }$ can be dropped because it has no effect on energy differences and transition rates. (Clearly the present non-relativistic formulation is not adequate to deal with the self-energy terms.) We can shift the Coulomb term from ${\hat{H}}^{\mathrm{rad}}$ to ${\hat{H}}^{\mathrm{matt}}$ and write $\hat{H}$ in the standard form,

(155) $$\hat{H}={\hat{H}}^{\mathrm{rad}}+{\hat{H}}^{\mathrm{mat}}\;,$$

with

(156) $${\hat{H}}^{\mathrm{rad}}=\int d^{3}x\;\frac{1}{2}\delta_{ab}\left({\hat{E}}_{\mathrm{tr}}^{a}{\hat{E}}_{\mathrm{tr}}^{b}+{\hat{B}}^{a}{\hat{B}}^{b}\right)\;,$$

(157) $${\hat{H}}^{\mathrm{mat}}=\sum_{n}\frac{-\hbar^{2}}{2m_{n}}\delta^{ab}D_{na}D_{nb}+\frac{1}{2}\sum_{n\neq n^{\prime }}\frac{q_{n}q_{n^{\prime }}}{4\pi |{\vec{z}}_{n}-{\vec{z}}_{n^{\prime }}|}\;.$$

We have reached familiar QED territory. Even though the ED ontic/epistemic interpretation differs from the standard textbook presentations, the ED equations reproduce the standard formalism of QED in the Coulomb gauge.

### 6.6. The Action

In the ED framework the introduction of an action principle is not fundamental; it is a convenient way to summarize the content of an already established formalism. The idea is to construct an action that reproduces the equations of motion and the constraints.

Consider a region *R* of the e-phase space and a time interval *T* that extends from $t_{1}$ to $t_{2}$, and define the action functional

(158) $$\begin{array}{cc} A[\Phi ,\psi ,\psi^{*}] & =\int_{RT}dt\;dzDA\;\psi^{*}\left(i\hbar \partial_{t}-{\hat{H}}_{\Phi }\right)\psi \\ \; & =\int_{RT}dt\;dzDA\;\psi^{*}\left(i\hbar \partial_{t}-\hat{H}\;-\int d^{3}x\;\Phi_{xt}\left({\hat{\rho }}_{x}^{e}-\partial_{ax}{\hat{E}}_{x}^{a}\right)\right)\psi \;. \end{array}$$

where $\tilde{H}$ is given in (65). The equations of motion and the Gauss constraint are obtained by requiring that the action be stationary under variations δψ and $\delta \psi^{*}$ that vanish at the boundary of the region RT. The functional $\Phi_{xt}\left[zA\right]$ is a Lagrange multiplier that enforces the Gauss constraint. Then

(159) $$\begin{array}{cc} \delta A= & \int_{TR}dt\;dzDA\;\left[\delta \psi^{*}\left(i\hbar \partial_{t}\psi -\hat{H}\psi -{\hat{\Gamma }}_{\Phi }\psi \right)-\left(i\hbar \partial_{t}\psi^{*}+{\hat{H}}^{*}\psi^{*}+{\hat{\Gamma }}_{\Phi }^{*}\psi^{*}\right)\delta \psi \right.\\ \; & \left.+\;\psi^{*}\int d^{3}x\;\delta \Phi_{xt}\left(\partial_{ax}{\hat{E}}_{x}^{a}-{\hat{\rho }}_{x}^{e}\right)\psi \right]=0. \end{array}$$

The $\delta \Phi_{xt}\left[zA\right]$ variation leads to the constraint

(160) $$\left(\partial_{a}{\hat{E}}^{a}-{\hat{\rho }}^{e}\right)\psi \approx 0\;\mathrm{or}\;{\hat{\Gamma }}_{\Phi }\psi \approx 0\;,$$

while $\delta \psi^{*}$ and δψ lead to

(161) $$i\hbar \partial_{t}\psi =\left(\hat{H}\;+{\hat{\Gamma }}_{\Phi }\right)\psi$$

and its complex conjugate.

## 7. Final Remarks

With the derivation of the QED Hamiltonian, whether in an unspecified gauge, Equation (130), or in the Coulomb gauge, Equation (155), the ED formulation of QED reaches a certain level of completion. On the basis of entropic inference we have not just derived electrodynamics; we have derived *quantum* electrodynamics. We have derived the linear Schrödinger equation with its attendant superposition principle. Along the way we have justified the need for complex numbers and the arrow of time. These are general consequences of the ED framework. Here we have expanded the framework to include invariance under local gauge transformations. This has allowed us to rederive charge conservation, the Maxwell equations, the Coulomb potential, and the familiar QED equations that have historically received so much empirical support. And, of course, the understanding of local gauge invariance within the ED setting is a necessary stepping stone towards relativistic quantum field theories such as Yang–Mills theories and gravity.

Much, however, remains to be done. Most glaring is the absence of any mention of the concept of photons. We have two reasons not to pursue those applications where the concept of photons becomes an indispensable tool. One reason is to avoid this paper becoming too long. Another reason is that the actual study of the quantum correlations between particles and fields, the transition rates of photon emission and absorption, and so much more can now be pursued by standard techniques. But there is one important remark concerning photons that must be included here.

Photons are the names we give to those special quantum states—the wave functions of certain excited states of the radiation field–that provide the indispensable vocabulary to analyze exchanges of energy and momentum. Therefore, even before one addresses the details of the construction of Fock spaces for the radiation field, the ED framework allows us to arrive at the following possibly unsettling conclusion: *photons are not particles and, even worse, they are not real*. The point being made is simple. The ED philosophy insists on a clear ontological commitment: particles and radiation fields are real, and they belong to the ontic sector. Probabilities and wave functions are not real, and they belong to the epistemic sector. They are tools we have developed to figure out what the real things might be doing—what the ontic microstates might be and how they might be changing. Perhaps surprisingly, quantities such as energies, linear and angular momenta, electric currents and even photons all belong to the epistemic sector. They are not properties of ontic particles or ontic fields but concepts that are useful in the analysis of the dynamics of probabilities. 

## Data Availability

Data is contained within the article.

## Abbreviations

The following abbreviations are used in this manuscript:

ED	Entropic Dynamics
QM	Quantum Mechanics
QED	Quantum Electrodynamics

## Appendix A. The Continuity Equation

The goal is to rewrite the integral equation for the evolution of probability, Equation (46), in differential form as a continuity equation. We proceed by adapting a technique [4] borrowed from diffusion theory [15]. Multiply Equation (46) by a smooth test functional $f_{z^{\prime }A^{\prime }}$ and integrate over $\left(z^{\prime },A^{\prime }\right)$ with measure $dz^{\prime }DA^{\prime }$,

(A1) $$\int dz^{\prime }DA^{\prime }\rho_{z^{\prime }A^{\prime }}^{\prime }f_{z^{\prime }A^{\prime }}=\int dzDA\left[\int dz^{\prime }DA^{\prime }\;P_{z^{\prime }A^{\prime }|zA}f_{z^{\prime }A^{\prime }}\right]\rho_{zA}\;.$$

The test functional $f_{z^{\prime }A^{\prime }}$ is assumed to be sufficiently smooth precisely so that orders of integration can be swapped and we can Taylor-expand about (z,A). Since Δz and ΔA are of $O(\Delta t^{1/2})$, keeping terms up to O(Δt), then as Δt→0, the integral in the brackets is

(A2) $$\begin{array}{cc} \left[\ldots \right] & =\int dz^{\prime }DA^{\prime }\;P_{z^{\prime }A^{\prime }|zA}\left(f_{zA}+\sum_{n}\frac{\partial f}{\partial z_{n}^{a}}\Delta z_{n}^{a}+\frac{1}{2}\sum_{nn^{\prime }}\frac{\partial^{2}f}{\partial z_{n}^{a}\partial z_{n^{\prime }}^{b}}\Delta z_{n}^{a}\Delta z_{n^{\prime }}^{b}\right.\\ \; & +\int d^{3}x\;\frac{\delta f}{\delta A_{ax}}\Delta A_{ax}+\frac{1}{2}\int d^{3}x_{1}d^{3}x_{2}\;\frac{\delta^{2}f}{\delta A_{ax_{1}}\delta A_{bx_{2}}}\Delta A_{ax_{1}}\Delta A_{bx_{2}}+\\ \; & +\sum_{n}\left.\int d^{3}x\;\frac{\partial }{\partial z_{n}^{a}}\left(\frac{\delta f}{\delta A_{bx}}\right)\Delta z_{n}^{a}\Delta A_{bx}\right)+o\left(\Delta t\right)\;. \end{array}$$

Rearrange this using Equations (49) and (51):

(A3) $$\begin{array}{cc} \left[\ldots \right] & =f_{zA}+\Delta t\sum_{n}\left(\left[\frac{\delta^{ab}}{m_{n}}\left(\partial_{nb}\varphi_{zA}-\beta_{n}A_{bz_{n}}\right)\right]\frac{\partial f}{\partial z_{n}^{a}}+\frac{1}{2}\frac{\partial^{2}f}{\partial z_{n}^{a}\partial z_{n}^{b}}\frac{\eta }{m_{n}}\delta^{ab}\right)\\ \; & +\Delta t\int d^{3}x\;c^{2}\delta_{ab}\left(\frac{\delta \varphi_{zA}}{\delta A_{bx}}\frac{\delta f}{\delta A_{ax}}+\frac{\eta }{2}\frac{\delta^{2}f}{\delta A_{ax}\delta A_{bx}}\right)\;. \end{array}$$

Substitute this into the RHS of (A1):

(A4) $$\begin{array}{cc} \mathrm{RHS} & =\int dzDAf_{zA}\rho_{zA}\\ \; & +\Delta t\int dzDA\sum_{n}\left(\left[\frac{\delta^{ab}}{m_{n}}\left(\partial_{nb}\varphi_{zA}-\beta_{n}A_{bz_{n}}\right)\right]\frac{\partial f}{\partial z_{n}^{a}}+\frac{\eta }{2m_{n}}\delta^{ab}\frac{\partial^{2}f}{\partial z_{n}^{a}\partial z_{n}^{b}}\right)\rho_{zA}\\ \; & +\Delta t\int dzDA\int d^{3}x\;c^{2}\delta_{ab}\left(\frac{\delta \varphi_{zA}}{\delta A_{bx}}\frac{\delta f}{\delta A_{ax}}+\frac{\eta }{2}\frac{\delta^{2}f}{\delta A_{ax}\delta A_{bx}}\right)\rho_{zA}\;. \end{array}$$

Next use

(A5) $$\begin{array}{cc} \int dz^{\prime }DA^{\prime }\rho_{z^{\prime }A^{\prime }}^{\prime }f_{z^{\prime }A^{\prime }}-\int dzDA\rho_{zA}f_{zA} & =\int dzDA\left(\rho_{zA}^{\prime }-\rho_{zA}\right)f_{zA}\\ \; & =\Delta t\int dzDA\frac{\partial \rho_{zA}}{\partial t}f_{zA}\;, \end{array}$$

integrate (A4) by parts, and divide by Δt to get

(A6) $$\begin{array}{cc} \int dzDA\frac{\partial \rho_{zA}}{\partial t}f_{zA} & =\int dzDA\sum_{n}\left(\frac{\partial }{\partial z_{n}^{a}}\left[-\rho_{zA}\frac{\delta^{ab}}{m_{n}}\left(\partial_{nb}\varphi_{zA}-\beta_{n}A_{bz_{n}}\right)\right]\right.\\ \; & +\left.\frac{\eta }{2m_{n}}\delta^{ab}\frac{\partial^{2}\rho_{zA}}{\partial z_{n}^{a}\partial z_{n}^{b}}\right)f_{zA}\\ \; & \int dzDA\int d^{3}x\;c^{2}\delta_{ab}\left(-\frac{\delta }{\delta A_{ax}}\left[\rho_{zA}\frac{\delta \varphi_{zA}}{\delta A_{bx}}\right]+\frac{\eta }{2}\frac{\delta^{2}\rho_{zA}}{\delta A_{ax}\delta A_{bx}}\right)f_{zA}\;. \end{array}$$

This must hold for arbitrary test functionals $f_{zA}$. Therefore we get a differential equation,

(A7) $$\begin{array}{cc} \frac{\partial \rho_{zA}}{\partial t} & =-\sum_{n}\frac{\partial }{\partial z_{n}^{a}}\left[\rho_{zA}\left(\frac{\delta^{ab}}{m_{n}}\left(\partial_{nb}\varphi_{zA}-\beta_{n}A_{bz_{n}}\right)-\frac{\eta }{2m_{n}}\delta^{ab}\frac{\partial \log\rho_{zA}}{\partial z_{n}^{b}}\right)\right]\\ \; & -\int d^{3}x\;c^{2}\delta_{ab}\frac{\delta }{\delta A_{ax}}\left[\rho_{zA}\left(\frac{\delta \varphi_{zA}}{\delta A_{bx}}-\frac{\eta }{2}\frac{\delta \log\rho_{zA}}{\delta A_{bx}}\right)\right]\;, \end{array}$$

which is a continuity equation for the probability flow $\rho_{zA}$ in the ontic configuration space

(A8) $$\frac{\partial \rho_{zA}}{\partial t}=-\sum_{n}\frac{\partial }{\partial z_{n}^{a}}\left(v_{n}^{a}\rho_{zA}\right)-\int d^{3}x\;\frac{\delta }{\delta A_{ax}}\left(u_{ax}\rho_{zA}\right)$$

with a current velocity (v,u) that has components along $z_{n}^{a}$,

(A9) $$v_{n}^{a}\left[z,A\right]=\frac{\delta^{ab}}{m_{n}}\left(\partial_{nb}\phi_{zA}-\beta_{n}A_{bz_{n}}\right)\;,$$

and along $A_{ax}$,

(A10) $$u_{ax}\left[z,A\right]=c^{2}\delta_{ab}\frac{\delta \phi_{zA}}{\delta A_{bx}}\;,$$

where

(A11) $$\phi_{zA}=\varphi_{zA}-\eta \log\rho_{zA}^{1/2}\;.$$
